# Coordination engineering for iron-based hexacyanoferrate as a high-stability cathode for sodium-ion batteries

**DOI:** 10.1073/pnas.2319193121

**Published:** 2024-07-25

**Authors:** Jiang Zhong, Lirong Xia, Song Chen, Zhengwei Zhang, Yong Pei, Hao Chen, Hongtao Sun, Jian Zhu, Bingan Lu, Yinghe Zhang

**Affiliations:** ^a^State Key Laboratory for Chemo/Biosensing and Chemometrics, College of Chemistry and Chemical Engineering, School of Physics and Electronics, Hunan Key Laboratory of Two-Dimensional Materials, Engineering Research Center of Advanced Catalysis of the Ministry of Education, Hunan University, Changsha 410082, People’s Republic of China; ^b^Department of Chemistry, Key Laboratory of Environmentally Friendly Chemistry and Applications of Ministry of Education, Xiangtan University, Xiangtan 411105, People’s Republic of China; ^c^Hunan Key Laboratory of Nanophotonics and Devices, School of Physics and Electronics, Central South University, Changsha 410083, People’s Republic of China; ^d^The Harold and Inge Marcus Department of Industrial Engineering, The Pennsylvania State University, State College, University Park, PA 16802; ^e^Shenzhen Research Institute, Hunan University, Shenzhen 518000, People’s Republic of China; ^f^School of Civil and Environmental Engineering, Harbin Institute of Technology, Shenzhen Key Laboratory of Advanced Functional Carbon Materials Research and Comprehensive Application, Shenzhen 518055, People’s Republic of China

**Keywords:** coordination engineering, iron-based hexacyanoferrate, Prussian blue analogs, sodium-ion batteries

## Abstract

Iron-based hexacyanoferrate (Fe-HCF) are promising cathode materials for sodium-ion batteries (SIBs) due to their unique open-channel structure that facilitates fast ion transport and framework stability. However, practical implementation of SIBs has been hindered by low initial Coulombic efficiency (ICE), poor rate performance, and short lifespan. Herein, we report a coordination engineering to synthesize sodium-rich Fe-HCF as cathodes for SIBs through a uniquely designed 10-kg-scale chemical reactor. Our study systematically investigated the relationship between coordination surroundings and the electrochemical behavior. Building on this understanding, the cathode delivered a reversible capacity of 99.3 mAh g^−1^ at 5 C (1 C = 100 mA g^−1^), exceptional rate capability (51 mAh g^−1^ even at 100 C), long lifespan (over 15,000 times at 50 C), and a high ICE of 92.7%. A full cell comprising the Fe-HCF cathode and hard carbon (HC) anode exhibited an impressive cyclic stability with a high-capacity retention rate of 98.3% over 1,000 cycles. Meanwhile, this material can be readily scaled to the practical levels of yield. The findings underscore the potential of Fe-HCF as cathodes for SIBs and highlight the significance of controlling nucleation and morphology through coordination engineering for a sustainable energy storage system.

## Significance

A critical challenge for iron-based hexacyanoferrate (Fe-HCF) cathode is that the extraordinary performance demonstrated in such materials was only achieved in ideal electrodes with perfect crystal structure and rather open framework for ion transport. This ideal crystal structure (without defect) is hard to accomplish in the practical preparation technology, especially existent the defect and coordinated water in a lattice framework. We design the Fe-HCF cathodes by adjusting the coordination environment. The half-cell of Na^+^ delivers a reversible capacity of 51 mAh g^−1^ at 10,000 mA g^−1^ and superior long-span cycling performance over 15,000th at 5,000 mA g^−1^. The full-cell exhibits long-term cycling at 500 mA g^−1^, with a capacity retention of 98.3% and a Coulombic efficiency maintaining 100% after 1,000th.

Sodium-ion batteries (SIBs) have been recognized as one of the most promising alternatives to traditional lithium-ion batteries, offering a solution to the issues of fossil energy overconsumption and limited lithium resources (1–4). Among the various cathode materials for SIBs, iron-based hexacyanoferrate (Fe-HCF), belonging to the Prussian blue analogs (PBAs) category, has gained attention due to its cost-effectiveness and electrochemical stability (5–7). However, before Fe-HCF cathodes can be utilized in grid-scale energy storage systems, two inherent drawbacks need to be addressed: sodium-poor state and high content of coordination water, which lead to a low initial Coulombic efficiency (ICE) and poor rate performance (5, 8, 9). Additionally, it is crucial to ensure a long operational lifespan while maintaining efficient energy storage capability (4). The primary factor contributing to the rapid degradation of the electrochemical performance is the distortion of the Fe-HCF framework, caused by the rapid growth of crystals during the synthesis process by conventional coprecipitation methods (6). So far, although some strategies have been proposed to adjust the preparation conditions to reduce the defect and coordination water in crystal structure, it is difficult to obtain satisfactory outcomes.

Fe-HCF, being coordination compounds, typically consist of metal cations (Fe^2+^) as nodes, which are connected by inorganic ligands (C≡N^−^) acting as bridges or linkers (5–8). The coprecipitation method, as the conventional approach to produce Fe-HCF, results in crystals that inevitably introduce abundant vacancies in structure due to Fe(CN)_6_^4−^ dissociation and recombination (10, 11). The crystal framework of Fe-HCF materials can be regulated by adjusting the synthesis conditions. As for Fe-HCF materials, the coordination surroundings (such as pH value, complexing agent, temperature, etc.) will affect the crystal growth during the synthesis process and will ultimately be reflected in the robustness of the crystal microstructure (9, 10). The sizes and shapes of Fe-HCF can be controlled by manipulating the synthetic conditions. Especially, the chelating agents could delay the node ion (Fe^2+^) reconstruction, which can influence the crystallization rate for Fe-HCF. Meanwhile, the concentration of Na^+^ in reaction solution can affect the content in the framework structure of Fe-HCF, ultimately determining the robustness of the crystal structure during the synthesis process (12). This inherent tunability makes the synthesis of Fe-HCF particularly attractive. Additionally, the electron interaction during self-assembly process of nodes and ligands will influence the sodium concentration and interstitial water contents (9–13). These factors ultimately decide the stability of structure and the electrochemistry behavior for Fe-HCF as cathode materials. Based on this understanding, it is meaningful to apply the principles of coordination chemistry to adjust the coordination environment in order to obtain high-quality Fe-HCF with good electrochemical performance. Nevertheless, finding the Archimedes’ pivot between appropriate coordination environments and ideal crystal structures is a key scientific challenge in the exploration of Fe-HCF materials to enhance the application of SIBs (11–13). Thus, understanding the crystal structure and coordination surroundings is pivotal for enhancing the overall performance as a Na-storage medium.

Previous attempts to regulate coordination surroundings to achieve a robust Fe-HCF structure with satisfactory electrochemical properties have yielded limited success. For instance, Goodenough and colleagues synthesized sodium-rich Fe-HCF using ascorbic acid to change the ligand by the hydrothermal method (10). Zhou proposed a low-pressure drying method to synthesize Fe-HCF with low content coordinated water as cathode for SIBs (14). Guo’s group introduced ethylene glycol as the solvent to reduce the content of coordinated water and prevented the oxidation of Fe^II^ to Fe^III^ in Fe-HCF simultaneously (15). Although many strategies based on the principle of coordination chemistry have been designed to enhance the electrochemical performance in Fe-HCF materials, there are still hard to acquire satisfactory Na-ions storage capabilities (16–18). Therefore, it is crucial to regulate and explore suitable synthesis conditions based on coordination engineering to understand the interaction between crystal structure and each synthesis conditions.

In this work, we proposed the concept of coordination engineering, which comprehensively considers the interactions between crystal structure and coordination surroundings. According to this concept, we developed a facile coprecipitation method based on coordination engineering to synthesize Fe-HCF (denoted NFCN) as cathodes for SIBs. By systematically regulating the coordination surroundings during coprecipitation, the NFCN materials with high reversible Na content and low coordinated water were obtained. The electrochemical behaviors of NFCN electrodes exhibited a reversible phase transformation during Na^+^ (de)intercalations, resulting in high sodium storage performance. Specifically, it can deliver a high ICE about 92.7% at a current density of 10 mA g^−1^, and 99.3 mAh g^−1^ under an increased current of 500 mA g^−1^. Meanwhile, it exhibits exceptional rate capability (51 mAh g^−1^ even at 100 C) and long cycling lifespan (over 15,000 times at 50 C). Furthermore, the full-cell shows a long-life span of over 1,000 cycles with high-capacity retention rate of 98.3%, highlighting the great potential of Fe-HCF cathode for practical applications in SIBs. Our work emphasizes the importance of a coordination environment for controlling the nucleation and morphology of NFCN, providing opportunities for utilizing inexpensive NFCN to build sustainable energy storage devices for grid-scale applications.

## Results

### Material Synthesis and Characterizations.

**Fig. 1.**
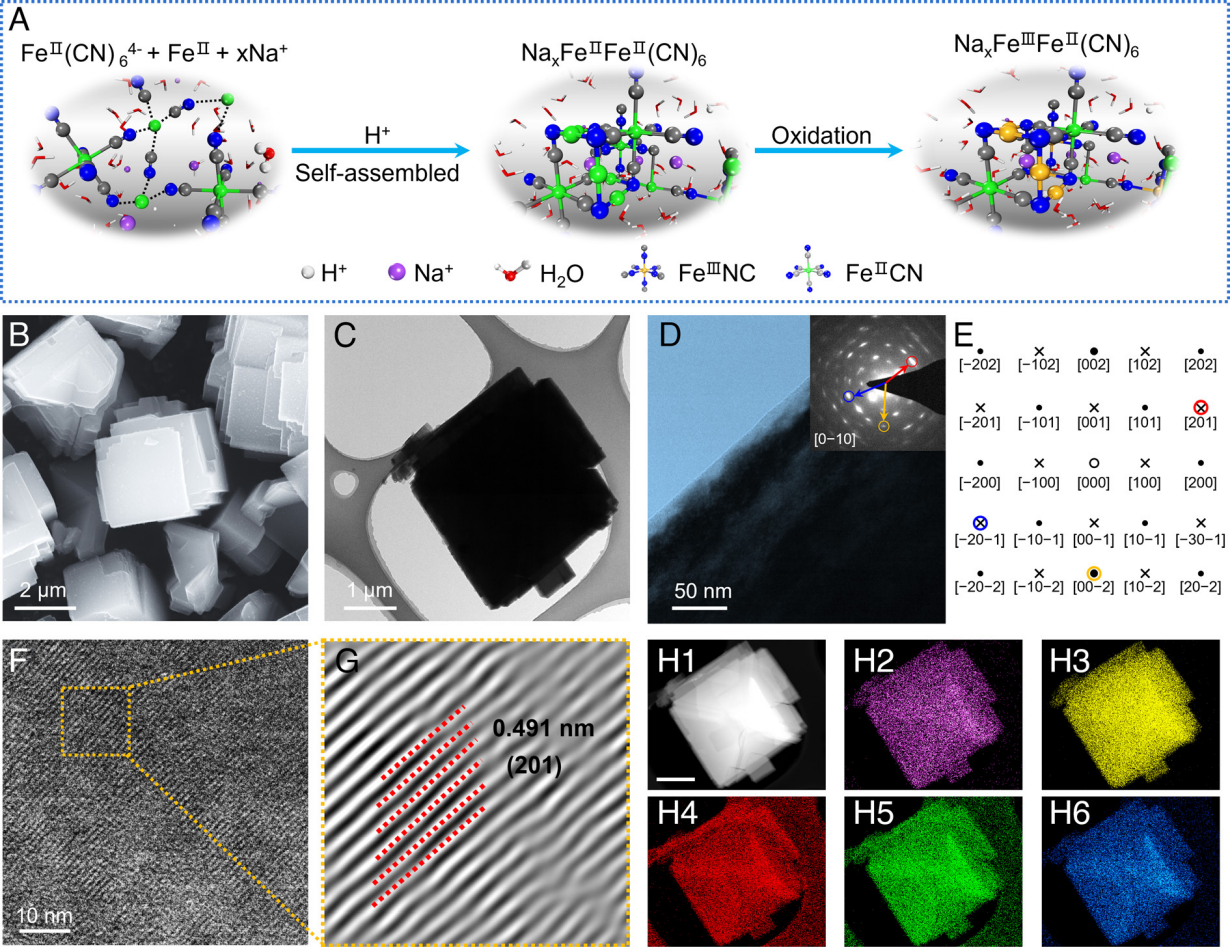
Influence of coordination environment on particle morphological control of NFCN samples. (*A*) Illustration of coordination engineered to drive molecule self-assembled. (*B*) SEM images of the NFCN-2 sample. (*C* and *D*) TEM image for NFCN-2 (*Inset* of *D*: the corresponding SAED patterns). (*E*) The simulated diffraction pattern of NFCN-2 along the [0-10] zone axis. (*F* and *G*) HRTEM for NFCN-2 and the corresponding lattice spacing. (*H*1–*H*6) EDS mapping of the different elements (purple: Na; yellow: Fe; red: C; green: N; blue: O) in the NFCN-2 sample (the scale bar for each of these images is 500 nm).

**Fig. 2.**
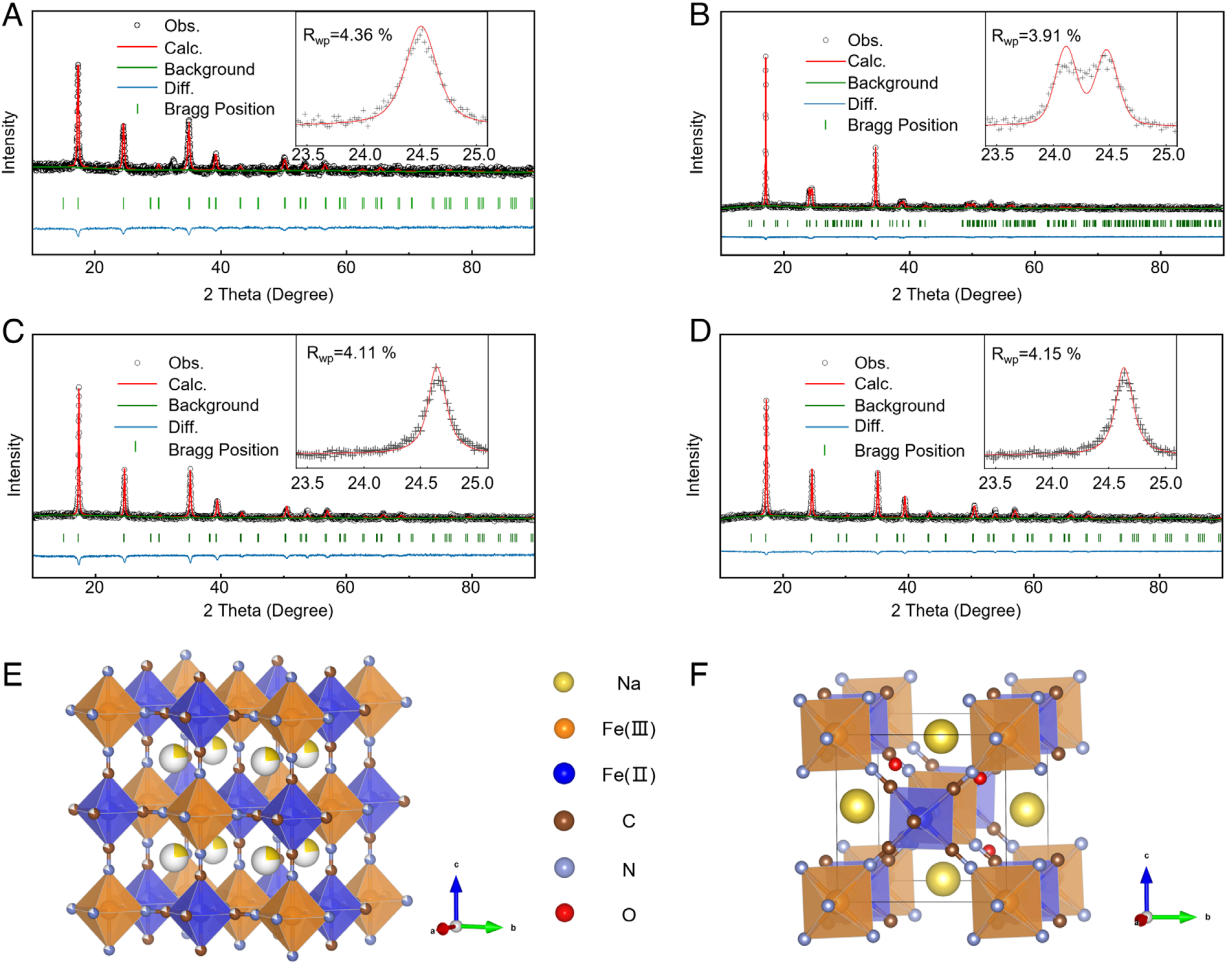
Structure and Rietveld refinement results. Rietveld refinements for NFCN-1 (*A*), NFCN-2 (*B*), NFCN-3 (*C*), and NFCN-4 (*D*). Schematic illustrations of the structures of the cubic NFCN-1, NFCN-3, and NFCN-4 (*E*) and monoclinic NFCN-2 (*F*).

In this study, we synthesized a series of sodium-based PBAs with diverse structures and morphologies using optimized coprecipitation methods grounded in coordination engineering. *SI Appendix,* Fig. S1 illustrates the chemical coprecipitation process to prepare all electrode materials obtained through a citrate-assisted coordination method using Na_4_Fe(CN)_6_ and FeSO_4_ precursors. Coordinate interactions drive various ions to self-assemble. The Fe^II^ will first dissociate in Fe(CN)_6_^4−^ and become a clincher to link the ferrocyanide ion in this process. However, it is inevitable that Fe^II^ will be oxidized to Fe^III^ due to exist oxygen during solution, resulting in the Fe-HCF structure being mutually arranged by Fe^II^ and Fe^III^ ion (5). Fig. 1*A* clearly shows that the Fe^II^ ion is linked to a carbon atom, while the Fe^III^ ion is bonded to a nitrogen atom (6). The driving force of coordination environment prompts Fe(CN)_6_^4−^ towards multistage coupling, leading to the production of a preframework of Fe-HCF. The controlled modulation of the coordination environment plays a crucial role in dictating the dissociation dynamics of Fe(CN)_6_^4−^ ions during the synthesis process. Exactly, the synthesis conditions designed through coordination engineering strike a balance between suitable coordination environments and ideal crystal structures. Therefore, by properly adjusting the synthesis conditions in accordance with the principles of coordination chemistry, such as solvent type, synthesis temperature, chelating agents, and pH value, we can precisely control these factors to further influence the crystal structure. This modulation not only impacts the rate and extent of dissociation but also influences subsequent multistage coupling reactions. For instance, the chelating agents can delay the reconstruction of node ions (Fe^2+^), which can influence the defect content in the crystal. The concentration of Na^+^ in reaction solution can affect the content in framework structure. The interplay between the coordination environment and the dissociation behavior underscores a nuanced and dynamic aspect of the overall electrochemical process, highlighting the importance of precise control over coordination conditions for tailoring the multistage coupling phenomena in the synthesized sodium-rich Fe-HCF cathode materials. The framework of Fe-HCF will evolve with even minor changes in the coordination environment. Indeed, different coordination environments were achieved by altering the pH value, producing four different samples, all denoted as NFCN. Scanning electron microscope (SEM) images are displayed in Fig. 1*B* and *SI Appendix*, Figs. S2−S5, for all samples. Specifically, the NFCN-1 sample revealed big aggregates with a dimension of around 6 μm. As the pH value decreased, the NFCN-2 sample displayed irregular polyhedral shapes with a reduced particle size of about 2 μm. As the reaction progressed, the strong acidity of H^+^ gradually etched the surface of the NFCN with dramatically reduced dimensions (Fig. 1*C* and *SI Appendix*, Fig. S5). Notably, when the pH value fell below 3.0, the particle size was reduced to approximately 100 nm, and the crystallization was suppressed toward a more amorphous phase, as indicated by the selected area electron diffraction patterns (Fig. 1*D* and *SI Appendix,* Fig. S5). According to the fast Fourier transform (FFT) pattern, the [201], [−201], and [00−2] planes of NFCN-2 are indexed and marked by different rings in red, blue, and yellow, respectively. The simulated diffraction pattern [using the tool cell-Viewer by CrysTBox software (19)] along the [0-10] zone axis of NFCN-2 is shown in Fig. 1*E*, where both the symmetry of pattern and d-spacings in reciprocal space are consistent with the experimental FFT pattern (20). Meanwhile, the high-resolution transmission electron microscopy (HRTEM) images show that the lattice fringes of NFCN-2 display interplanar spacings of 0.491 nm, which match well with the (201) plane (Fig. 1 *F* and *G*). In addition, we measured the lattice spacing of the *P2_1_/n* space group NFCN-2 particle using FFT and inverse FFT (*SI Appendix,* Fig. S6), which is consistent with the selected area electron diffraction (SAED) results. Overall, the morphology changed from large-sized aggregates to irregular-shaped microcubes and finally to nano-cubes, depending on the different coordination environments. Their corresponding energy-dispersive spectrometer (EDS) analysis was used to detect the evenly elemental distribution (Na, Fe, C, N, and O elements), and the results are shown in Fig. 1*H* and *SI Appendix*, Figs. S2−S4. The crystallographic information of these samples was explored in detail through Rietveld refinement using GSAS-II software, such as atom locations and occupations (see the part of *Materials and Methods* for more information) (21). In Fig. 2 *A*−*D*, it can be observed that all samples (e.g., NFCN-1, NFCN-3, and NFCN-4) demonstrated a cubic phase with $Fm\bar{3}m$ space group, except for NFCN-2 that showed an unusual monoclinic structure with *P2_1_/n* space group. In particular, distinct cubic and monoclinic phases are demonstrated in the schematics for samples NFCN-1, NFCN-3, and NFCN-4 (Fig. 2*E*) and NFCN-2 (Fig. 2*F*) due to the different synthesis parameters. The refinement results on the precipitation process and lattice parameters are provided in *SI Appendix,* Tables S1–S4. Additionally, the element concentration (Na and Fe) and coordination water content for all samples were confirmed using inductively coupled plasma-optical emission spectroscopy (ICP-OES) and thermogravimetric analysis (TGA), respectively, as shown in *SI Appendix,* Fig. S7 and Table S5. The corresponding chemical formulas are summarized in *SI Appendix,* Table S1.

Before precipitation, the as-prepared FeSO_4_/sodium citrate solution should be in a suitably acidic condition to keep the stable, coordinated surroundings for Fe^2+^. The chelating ability of metal ions is strongly affected by pH value due to the hydration of metal ions that decreases with increased acid effect, thereby reducing the stability of the metal complex. The X-ray photoelectron spectroscopy (XPS) results indicate that increased acidity has led to the oxidation of the Fe^2+^ to Fe^3+^ in NFCN-3 and NFCN-4 samples (*SI Appendix,* Fig. S8) (20, 21). Therefore, carefully controlling the coordination environment according to the principle of coordination chemistry makes it possible to synthesize particles with various crystallites, morphologies, and compositions. Although the reduced dimensions of our nanostructured NFCN samples can enhance the specific capacity of the NFCN electrode, it can also lead to significant side reactions occurring at the interfaces between the electrode and electrolyte, as well as low volumetric energy density. For practical battery applications, microsized particles are ideal as electrode materials due to their high tap density and low surface area, which leads to high volumetric performance and reduced side reactions (21). The Brunauer−Emmett−Teller (BET) and the Barrett−Joyner−Halenda (BJH) analysis, as demonstrated in *SI Appendix,* Figs. S9 and S10 and Table S6, indicate that NFCN-2 has a lower specific surface area (15 m^2^ g^−1^) and fewer pores than other NFCN samples.

### Electrochemical Evaluation of all NFCN Samples.

**Fig. 3.**
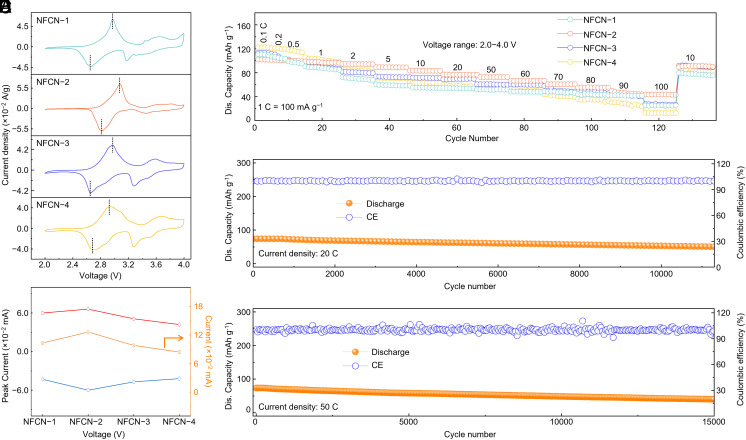
Electrochemical characteristics of various NFCN electrodes for half-cell SIBs. (*A*) CV curves at a scan rate of 0.1 mV s^−1^. (*B*) Current gaps between anodic and cathodic peaks and peak current of the CV curve of various samples. (*C*) Rate performance at different current densities from 0.1 to 100 C. (*D* and *E*) Long-term cycling at 20 and 50 C.

According to previous research, we conducted a comprehensive examination to assess the impact of various synthesis conditions, derived from coordination engineering strategies, on the electrochemical performance of Fe-HCF. To correlate crystal structures with electrochemical properties, we have conducted a thorough XRD characterization and Rietveld refinement of the Fe-HCF materials. As detailed in Fig. 2 and the *SI Appendix*, Figs. S11–S13 and Tables S2 and S7–S9, it can be observed that the introduction of chelating agents significantly affects the crystalline phase of Fe-HCF. Whereas, the temperature and solvent environment primarily influence the lattice parameters (e.g., the lattice tends to shrink at elevated temperatures). Additionally, adjusting the pH has been found to impact both the crystalline phase and lattice parameters simultaneously. Using these insights, we evaluated the electrochemical performance of NFCN cathodes at consistent mass loadings. *SI Appendix,* Figs. S14–S16 present the electrochemical performance for Fe-HCF at different synthesis conditions. After a systematic comparison, we have identified the optimal coordination conditions that determine the formation of ideal crystal structures. These conditions include suitable solvent surroundings, moderate temperature, appropriate pH value and complexing agents. Based on the results, we selected the optimal synthesis condition to fabricate Fe-HCF materials. The cyclic voltammetry (CV) curves are demonstrated in sodium-ion half-cells (Fig. 3*A*). NFCN-3 and NFCN-4 cathode exhibited low current density response among the four electrodes, indicating poor energy storage capability (22–24). Comparing the current density gap between oxidation and reduction peaks illustrates an increasing trend and a decrease from NFCN-1 to NFCN-4 (Fig. 3*B*), exhibiting an analogous volcano-shaped curve (22). NFCN-2 demonstrates the highest peak current density difference between to anodic and cathodic peaks, which exhibit higher activity than other electrodes under the same conditions (22). And the order from great to small of energy storage performance degree was NFCN-4, NFCN-3, NFCN-1, and NFCN-2 at 0.1 mV/s. These values align with the electrochemical performance (*SI Appendix,* Fig. S17). Indeed, the NFCN-2 electrode also delivered a much better performance at various current densities from 10 to 10,000 mA g^−1^ (denoted as 0.1 to 100 C), especially at the high current densities, compared to the other electrode counterparts (Fig. 3*C*). Especially, the NFCN-2 cathode can reach a reversible capacity of 51 mAh g^−1^ even at an ultrahigh rate of 100 C (10 A g^−1^). This means that the NFCN-2 cathode in a half-cell SIBs can be fully charged within just about half a minute. The high-rate capability of the NFCN-2 cathode is mainly due to its open structure for fast ion transport (25). All electrodes demonstrated excellent reversibility when restoring to the relatively low current density. To the best of our knowledge, the high-rate performance of NFCN-2 is the best among the reported PBA-based cathode materials for SIBs (6, 8, 9, 15, 17, 20, 21, 23, 26, 27). Furthermore, the long-term cycling performance of the NFCN-2 electrodes was compared at different current densities, as shown in Fig. 3 *D* and *E*. The NFCN-2 electrode delivered 85.5 mAh g^−1^ with capacity retentions of ~70% after 10,000 cycles at 20 C. To further highlight the NFCN-2 electrode, long-cycle performance tests at specific currents of 50 C (Fig. 3*E*) were performed. The NFCN-2 preserves a highly stable coulombic efficiency and long lifespan (reaching to 15,000 cycles).

### Investigation of Electrochemical Kinetic and Sodium-Ion Diffusion Behavior.

**Fig. 4.**
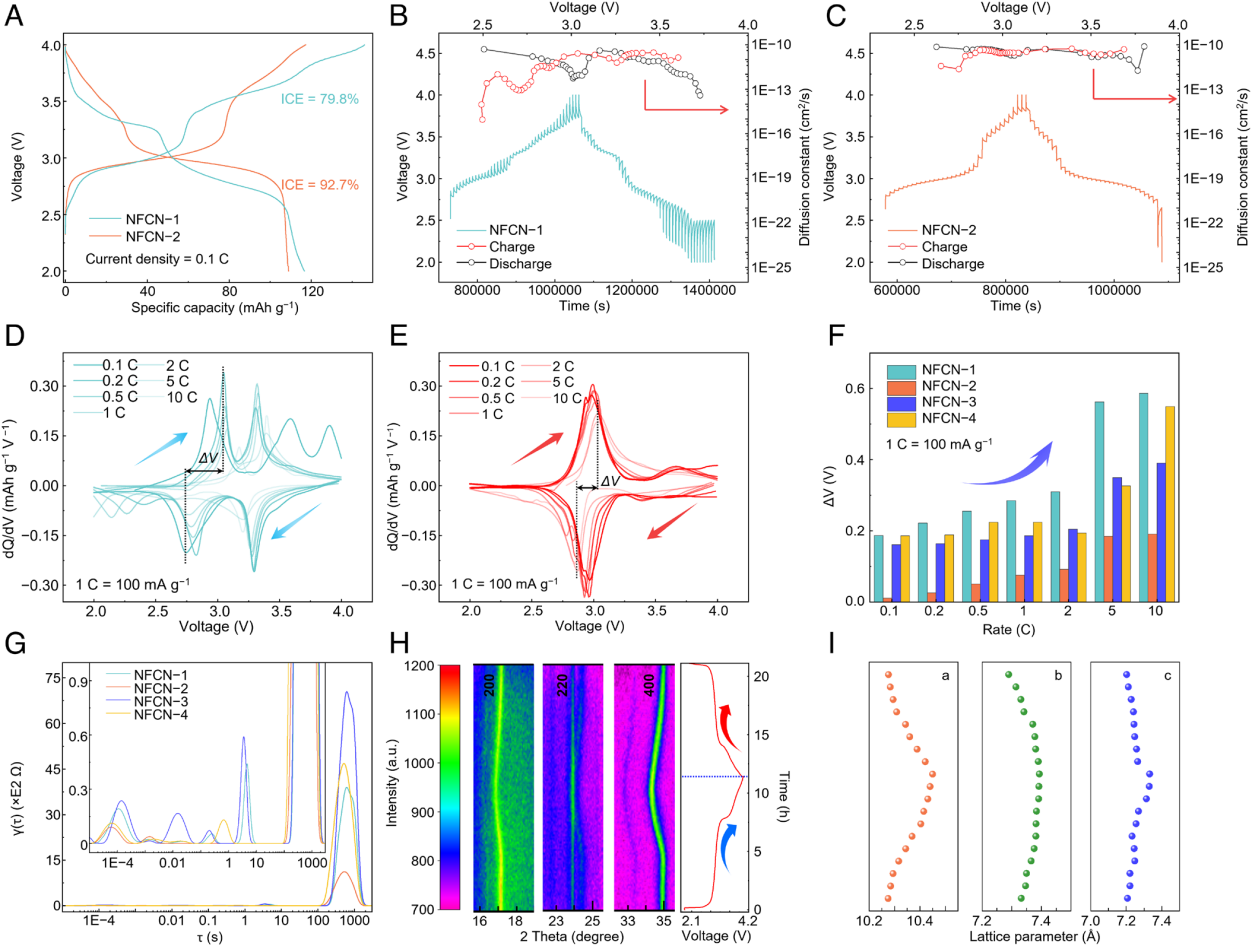
Electrochemical kinetic studies for various NFCN electrodes. (*A*) The initial charge-discharge curves for NFCN-1 and NFCN-2 electrodes. (*B* and *C*) The GITT curves and calculated diffusion constant for the NFCN-1 and NFCN-2 electrodes, respectively. (*D* and *E*) The d*Q*/d*V* curves of NFCN-1 and NFCN-2 electrode testing at different rates (0.1 to 10 C) at the voltage window of 2.0 to 4.0 V, respectively. (*F*) The plot of Δ*V* as a function of the rate. Δ*V* is the voltage difference of the charge and discharge peak in the d*Q*/d*V* curves to quantify the polarization. (*G*) The DRT transformation obtained from EIS results unravels the charge transfer evolution. (*H*) Intensity bar, color-mapped curves for in situ XRD patterns and the corresponding charge/discharge curves. (*I*) The changes of lattice parameters of NFCN-2 calculated from XRD results.

As shown in Fig. 4*A* and *SI Appendix,* Fig. S18, the discharge capacities for four various electrodes are around 120 mAh g^−1^, but the charge capacities vary from 140 mAh g^−1^ for the NFCN-1 electrode, 112 mAh g^−1^ for the NFCN-2 electrode, and about 59 mAh g^−1^ for both NFCN-3 and NFCN-4 electrodes. Thus, the initial Coulombic efficiencies (ICE) are calculated to be 79.8%, 92.7%, 187.6%, and 204%, respectively. Notably, the ICE of NFCN-3 and NFCN-4 electrodes exceeds 100%, indicating abnormal phenomena for cathode materials. The ideal cathode materials should have an ICE close to 100%, which is the critical parameter to evaluate the performance of SIBs (27, 28). The abnormal ICE for cathode materials is probably due to the sodium-poor host structure; meanwhile, it would be affected by active unite oxidation (Fe^II^), which is consistent with the high-resolution XPS results. Thus, to obtain a reversible NFCN cathode with high ICE, the high sodium content and the low oxidation state are desirable features that can be regulated by the coprecipitation synthesis. Moreover, the plateau around 3 V of NFCN-1 is much shorter than that of NFCN-2. A long and gradual plateau indicates a large quantity of active ion deintercalation and a low degree of polarization, vice versa. In other words, the short plateau can be attributed to the low-sodium content in the electrode material (18).

The Galvanostatic intermittent titration technique (GITT) test was conducted to analyze the electrochemical kinetic. The fresh coin cell was alternately charged for 10 min followed by 60 min resting and then discharged similarly (29, 30). Typical GITT curves of various NFCN cathodes are shown in Fig. 4 *B* and *C* and *SI Appendix,* Fig. S19. The calculations and mathematic derivations were elaborated in Supplementary text. The calculated diffusion coefficient for Na^+^ (*D*_Na+_) of various cathodes is close due to the same open-channel structure, and *D*_Na+_ of NFCN-2 cathode material is slightly higher, which might be due to a more stable sodium-rich monoclinic structure. The more ion transports tend to be accompanied by higher potential polarization during cell operation, except for the favorable crystal framework, which provides sufficient space to fulfill charge delivery. Fig. 4 *D* and *E* and *SI Appendix*, Figs. S20 and S21 present the corresponding differential capacitance curve (d*Q*/d*V*) and charge/discharge curves at different rates. The voltage gap (Δ*V*) is the difference between anodic and cathodic peaks, usually identified to quantify the degree of polarization during charge-discharge at different current densities. As expected, the NFCN-2 electrode has lower polarization for a distinct structure. Fig. 4*F* exhibited the Δ*V* value for all samples, NFCN-2 is only 0.19 V at 10 C, indicating a lower polarization level than other NFCN cathodes (27).

To gain more insights into the kinetics, we conducted timescale-based studies to examine the rate of the mass transfer process (31, 32). Initially, electrochemical impedance spectroscopy (EIS) was performed (*SI Appendix,* Fig. S22), followed by the conversion of EIS spectra into a distribution of relaxation times (DRT), as depicted in Fig. 4*G*. The presence of peaks at specific relaxation times signifies corresponding kinetic processes, with peak areas reflecting impedance values. Relaxation processes within the range of 10^−4^ to 10^−2^ s represent the electrode’s interface response, associated with the formation of the solid electrolyte interphase (32). Values of τ below 10^−2^ s correspond to the charge transfer process (32–34). The NFCN-2 electrode displays two prominent peaks representing two charge transfer processes, whereas the other cathodes show a greater number of peaks, indicating more complex mass transfer processes (32–34). Meanwhile, an in situ XRD test was performed to investigate the structural evolution of the NFCN-2 cathode material, and the corresponding results are shown in Fig. 4*H*. The peaks observed at 17.2°, 24.5°, and 34.9° exhibit a gradual shift toward lower angles, indicating lattice expansion during Na^+^ deintercalation in NFCN-2. The slight peak shifts suggest minimal volume changes during the discharge process. During the subsequent discharging, the peaks shift toward higher angles and return to their original positions after reaching a full discharge of 2.0 V, highlighting a highly reversible phase change. The lattice parameters during charge and discharge, as summarized in Fig. 4*I*, demonstrated a high reversiblity of NFCN-2 structure. The in situ Raman is in accord with in situ XRD result, *SI Appendix,* Fig. S23 demonstrating the in situ Raman spectra for the NFCN-2 electrode. Since the C≡N^−^ group is sensitive to its surrounding chemical environment, when charged to 4.0 V, two peaks will emerge at ~2,090 and 2,120 cm^−1^. These two peaks can be ascribed to the Fe^III^-C≡N-Fe^III^ groups. After following the discharge process, the peak intensity is gradually decreased, suggesting that the Fe^III^ is reduced to Fe^II^ during the insertion of sodium-ion process. The slight variance in peak intensities can be assigned to the irreversible insertion/extraction of partial Na^+^ in the framework during the charging/discharging process. Furthermore, the structural change in NFCN-2 during operation is highly reversible, which is due to the absence of recognizable Raman shift (35). These findings demonstrate the superior structural stability of NFCN-2, enabling an extended cycle life. These kinetic characterizations agree with the rate performance of different NFCN electrodes.

The diffusion mechanism of the NFCN framework was investigated using the ab initio molecular dynamics (AIMD). According to the XRD refinement results, all NFCNs, except NFCN-2, exhibited a cubic phase with $Fm\bar{3}m$ space group, so we only selected NFCN-1 and NFCN-2 to carry out AIMD simulation of 50 ps at 900 K after optimization. As shown in *SI Appendix,* Fig. S24*A*, the energy with the change of time is almost invariable in the NFCN-2 system; however, the NFCN-1 system has drastic fluctuation, demonstrating the NFCN-2 with higher stability than NFCN-1. Additionally, *SI Appendix,* Fig. S24*B* exhibited the mean-square displacement (MSD) of Na^+^ over time. The MSDs in both the NFCN-1 and NFCN-2 system show a linear increase with time at 900 K, indicating that both the NFCN-1 and NFCN-2 structures facilitate the pathways for Na^+^ transport (21, 26). Moreover, the steeper MSD curve suggested faster Na^+^ diffusion in the NFCN-2 system, resulting in higher conductivity compared to the NFCN-1 system (the conductivity of NFCN-2: 51.64 mS cm^−1^; NFCN-1: 31.17 mS cm^−1^). For a visual representation of the sodium-ion diffusion pathway, we utilized the Pymatgen code for the calculation, and the corresponding Na^+^ probability density distribution is presented in *SI Appendix,* Fig. S24 *C* and *D*, which can display the areas where Na atoms can be found more frequently (36). Due to the wider diffusion channels in NFCN, Na^+^ diffuses rapidly, and the diffusion kinetics are essentially isotropic. Notably, the diffusion kinetics of the NFCN-2 system demonstrate a more symmetrical distribution, indicating faster Na^+^ diffusion, which can significantly enhance the electrochemical kinetics (26, 36).

### Large-Scale Production of NFCN and Electrochemical Performance of the Full Cell.

**Fig. 5.**
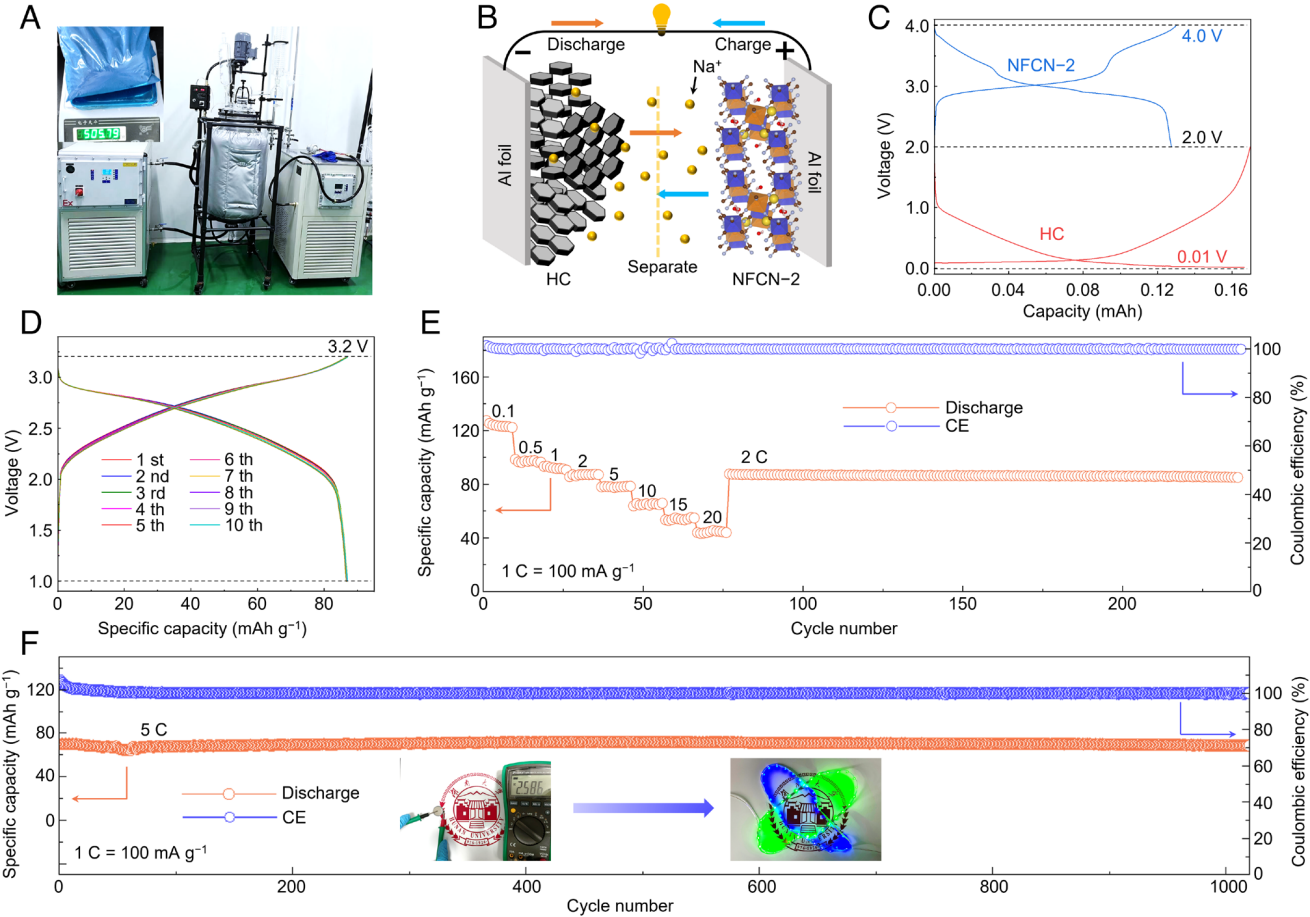
Large-scale production of NFCN-2 powder and electrochemical performance of the SIB full cell (NFCN-2|HC). (*A*) Demonstration of large-scale production of NFCN in a 100 L reactor (the insert is 0.5-kg powder of NFCN-2). (*B*) Working mechanism of the full cell. (*C*) Charge/discharge curves of the NFCN-2 and HC electrodes to determine the mass ratio for the full cell. (*D*) Charge/discharge curves at 2 C of the full cell. (*E*) Rate capability at different current densities from 0.1 C to 20 C. (*F*) Cycling performance of the full cell after 1,000 cycles at 5 C. The *Inset* figures display the open-circuit potential values for the presodiated NFCN-2|HC full cell and demonstrate its ability to power an LED flexible rope light.

Considering the practical application of NFCN-2 cathode material in SIBs, the mass production was carried out using the synthesis conditions optimized through coordination engineering in a 100 L reactor (Fig. 5*A*). To evaluate the practical applicability of the monoclinic NFCN-2 cathode, we conducted full-cell testing using NFCN-2 as the cathode material and hard carbon (HC) as the anode material and the working mechanism of the full cell is illustrated in Fig. 5*B*. The performance of the commercial HC electrode is presented in *SI Appendix,* Fig. S25. The voltage window was set at 1.0 to 3.2 V based on previous reports (7, 8, 14), and the negative to positive electrode ratio (N/P ratio) was approximately 1.3 to optimize the utilization of both cathode and anode capacity (Fig. 5*C*). As shown in *SI Appendix,* Fig. S26, the full cell exhibit an ICE of 101.4% for the 1st cycle and 100% for the the 2nd cycle at 0.1 C, indicating a side reaction occurring in the 1st cycle. Subsequently, the first 10 cycles for the full cell achieved a specific capacity of 87.3 mAh g^−1^ based on the mass of the cathode material at 2 C (Fig. 5*D*). Additionally, the full cell exhibited reversible capacities of 98.5, 92.1, 87.3 mAh g^−1^ at various rates of 0.5, 1, and 2 C, respectively, and maintained a total capacity of 45.5 mAh g^−1^ at a high rate of 20 C (Fig. 5*E*). Furthermore, the monoclinic NFCN-2 cathode material demonstrated long-term cycling at 5 C, with a capacity retention of 98.3% and an average coulombic efficiency of approximately 100% over 1,000 cycles (Fig. 5*F*). Meanwhile, the open-circuit potential (OCP) for the fresh presodiation full cell is approximately 2.5 V (*SI Appendix,* Fig. S27), and it can power an LED soft rope light (*Inset* of Fig. 5*F*). To the best of our knowledge, the long-term cycling performance of the NFCN-2||HC full cell is the longest reported among PBA-based cathode materials for full-cell SIBs (*SI Appendix,* Table S10).

## Discussion

In this study, guided by the principles of coordination engineering, the sodium-rich Fe-HCF cathode material has been successfully fabricated using the coprecipitation method. The optimized monoclinic NFCN-2 cathode exhibits noteworthy electrochemical characteristics, including a reversible capacity of 99.3 mAh g^−1^ at 500 mA g^−1^, remarkable rate capability (51 mAh g^−1^ at 10 A g^−1^), prolonged cycling stability (over 15,000 cycles at 50 C), and an elevated ICE of 92.7%. When coupled with a HC anode, it displayed an enduring cycling performance (over 1,000 cycles) with superior Coulombic efficiency (approximately 100%) and outstanding capacity retention (exceeding 98%). These findings underscore the potential of Fe-HCF as a cathode material for high-performance SIBs and emphasize the pivotal role of coordination engineering in tailoring crystal structure, nucleation, and morphology to achieve superior electrochemical performance in SIBs.

## Materials and Methods

### Synthesis of NFCN Samples.

All chemicals for synthesizing NFCN samples in the laboratory were purchased from Sinopharm Chemical Reagent limited corporation and used directly without further treatment. All samples were synthesized via a modified precipitation method at 25 °C. In a typical process, NFCN-1: 0.03 mol sodium citrate (C_6_H_5_O_7_Na_3_), 6 mmol FeSO_4_·7H_2_O, and 5 mL of hydrochloric acid (37 wt% solution in water) were mixed in deionized water, and a 100 mL solution was prepared, labeled as solution A. Then, 0.03 mol C_6_H_5_O_7_Na_3_ and 4 mmol Na_4_Fe(CN)_6_·10H_2_O were dissolved in another 100 mL solution labeled solution B. Both solutions were bubbled with Ar to protect Fe^2+^ from oxidation. Solution A was stirred for 3 h before use. Solutions A and B were transferred quickly into a constant pressure drop funnel and a three-neck flask (under Ar), respectively. Then, solution A was added dropwise into solution B, and the mixture was stirred continuously at 800 rpm for 6 h and aged for a few hours before centrifugation; the precipitate was washed with deionized water and ethanol three times, respectively, and finally, the wet powder was dried in a vacuum oven at 120 °C for 12 h. All samples were synthesized by the same route. The hydrochloric acid in solutions A was 8, 10, and 15 mL for NFCN-2, NFCN-3, and NFCN-4, respectively. Additionally, other varying synthesis conditions, such as temperature, complexing agents, and solvents etc., were explored using the same method. All chemicals for producing NFCN samples in the 100 L reactor were purchased from Xianglu Scientific Instrument (Hunan) Co., Ltd., China.

### Material Characterizations.

The obtained NFCN samples were investigated by powder XRD analysis (SmartLab 3 kW Rigaku, Japan) equipped using Cu kα radiation. The content of Na and Fe elements in all NFCN samples was identified by ICP–OES analysis (Agilent720ES Optical Emission Spectrometers, America) and element analyzer (Vario EL Cube), and the relative SD of the measured samples was less than 1.5%. SEM images were performed by Mira3 TESCAN (Czech Republic). Transmission electron microscopy (TEM) images were obtained by Titan S/TEM FEI (America). The pH value was measured via FE22-Standard (METTLER TOLEDO, Switzerland). XPS was taken on an ESCALAB250Xi (Thermo Scientific, America) spectrometer equipped with X-ray source (*hv* = 1,486.6 eV, monochromatic Al Ka). Nitrogen adsorption–desorption isotherm at 77 K was measured on an ASAP 2020 absorption analyzer (Micromeritics, America). The specific surface area and the pore size distribution of the samples were tested using the BET and the BJH analysis methods, respectively. TGA (PerkinElmer STA 8000) was conducted in the air atmosphere from room temperature to 600 °C at a heating rate of 10 °C min^−1^.

### Electrochemical Measurements.

#### Preparation of cathode.

The NFCN electrode slurry was prepared by mixing active material, conductive carbon, and polyvinylidene fluoride with a mass ratio of 7:2:1 dispersed in N-methyl pyrrolidone. Then, the slurry-coated electrodes were vacuum dried at 60 °C for 12 h, and the active mass loading was 1.2 to 1.5 mg cm^−2^.

#### Preparation of anode.

The HC (Type-2, Kuraray China Co., Ltd.) was mixed with water (0.725 mL), absolute ethyl alcohol (0.125 mL), and sodium carboxymethylcellulose (CMC) binder. Then, the prepared slurry was coated onto aluminum collectors. The typical electrode was made with a formula of active material, conductive carbon, and CMC with a mass ratio of 8:1:1. Then, the electrodes were vacuum dried at 80 °C for 12 h, and the mass loading of the active material was 1.0 to 1.5 mg cm^−2^.

#### Electrochemical tests.

CR2032 sodium-ion coin cells were fabricated inside an Ar-filled glovebox (MBRAUN UNIlab Plus, Germany) (water and oxygen content below 0.5 ppm) with 1 M NaClO_4_ in diethyl carbonate (DEC) and ethylene carbonate (EC) and fluoroethylene carbonate (FEC) (EC/DEC + 5% FEC, 1:1 vol/vol, Nanjing Mojiesi Energy Tech.) electrolyte (water content <10 ppm), a metallic sodium counter and reference electrode, and glass fiber (Whatman, GF/D). The charge–discharge test was carried out on a Land CT2001A battery cycler (LANDTE Co., Wuhan, China) and tested in a room temperature (25 ± 1 °C). Cyclic voltammetry (CV) and alternating-current impedance measurements (in a frequency range between 100 kHz and 0.001 Hz at potentiostatic signal amplitude of 10 mV) were performed using a Vertex.One.EIS electrochemical station (IVIUM Technologies BV). The simulation of the experimental impedance was conducted with IVIUM software. The equivalent circuit for the fitting of impedance spectra at the SOC of 0% was conducted by the generalized finite length Warburg element (W) for descriptive purposes.

### Calculation of Diffusion Coefficient.

The diffusion evolution is tracked based on the GITT. The following simplified equation evaluates the diffusion coefficient based on Fick’s law:

$$D_{\mathrm{GITT}}=\frac{4}{\pi \tau }{\left(\frac{n_{m}V_{m}}{S}\right)}^{2}{\left(\frac{{\Delta E}_{s}}{{\Delta E}_{t}}\right)}^{2},$$

where *τ* represents the relaxation time, *n*_m_ represents the mole value, and *V*_m_ is the mole volume, *S* is the contact area of electrode/electrolyte, Δ*E_s_* is the voltage response stimulated by the pulse current, and Δ*E_t_* is the voltage change by the galvanostatic discharge (34).

### Identification of DRT Peaks.

The DRT was calculated by the code of Python, which is demonstrated by Ciucci et al. (33, 37). The detailed processing step is as follows: first, using alternating-current impedance tester to obtain the impedance spectrum (in a frequency range between 100 kHz and 0.001 Hz); second, based on the open access Pycharm community software to operate the code of GP-DRT (33, 37, 38); finally, load the impedance spectrum data to train database.

### Rietveld Refinement Details.

The crystal structure of all NFCN samples was analyzed by GSAS II software (21). And the result is a face-centered cubic structure of Prussian blue (JCPDS No. 52-1907). Whereafter, the KNi(Co(CN)_6_)(H_2_O)_9.6_ (COD No. 1526335) was selected as the initial model to do the Rietveld refinement. The background, scale factor, zero, cell, and profile parameters, such as U, V, and W of the pseudo-Voigt function, were refined. Then, the O atom positions and thermal parameters were also refined until the convergence was achieved. The final reliability factors are shown in *SI Appendix,* Tables S2–S4, indicating that the refinement is credible.

### Calculation Method.

TheVienna ab initio Simulation Package (VASP) (39) was used to implement all the DFT calculations, utilizing the projector augmented wave pseudopotential method (40). The exchange-correlation energy was described by the function of Perdew−Burke−Ernzerhof (41), including van der Waals corrections (DFT-D3 method) (42). The kinetic energy cutoff of the electron wave functions was set as 520 eV. The geometry optimizations were performed using the conjugated gradient method, and the convergence thresholds were set at 10^−5^ eV for energy and 0.02 eV Å^−1^ for force. The Brillouin zone was sampled by the 3 × 3 × 1 and 3 × 3 × 2 Monkhorst−Pack scheme (43) for NFCN-1 and NFCN-2, respectively. To deal with the strongly correlated d-electrons, the rotationally invariant Dudarev method (DFT+U) was applied, and the U_eff._ for Fe was set to 4.0 eV (44). AIMD simulation was used to study the structure’s stability and the Na^+^ diffusion behavior. The cutoff energy was set as 220 eV, and the convergence thresholds were set at 10^−4^ eV for energy and 0.02 eV Å^−1^ for force. The AIMD of 50 ps for all the NFCN systems performed under the NVT ensemble, with a step length of 2 fs, temperature of 900 K, and a Nosé–Hoover thermostat, was chosen. Visualization of the electrolyte structures was carried out by using a VESTA (45).

### The MSD.

$${\left(\vec{r}\left(t\right)\right)}^{2}=\frac{1}{N}\sum_{i=1}^{N}\left({\left({\vec{r}}_{i}\left(t+t_{0}\right)\right)}^{2}-{\left({\vec{r}}_{i}\left(t_{0}\right)\right)}^{2}\right),$$

where ${\left(\vec{r}\left(t\right)\right)}^{2}$ is the MSD, the unit is Å^2^, *N* is the number of atoms, ${\vec{r}}_{i}\left(t+t_{0}\right)$ is the displacement at the *t_0_* point, and ${\vec{r}}_{i}\left(t_{0}\right)$ and is the reference position. And the diffusion coefficient is based on the equation:

$$D=\underset{t\to \infty }{lim}\left[\frac{1}{2dt}{\left(\vec{r}\left(t\right)\right)}^{2}\right],$$

where *D* is the diffusion coefficient, *d* is the dimensionality, and *t* is time.

The conductivity was measured using Pymatgen’s Diffusion Analyzer, the detailed guideline is already upload to https://pypi.org/.

## Supplementary Material

Appendix 01 (PDF)

## Data Availability

All study data are included in the article and/or *SI Appendix*. The data that support the findings of this study are available at GitHub (46).
