# Supplementary material for: Coordination engineering for iron-based hexacyanoferrate as a high-stability cathode for sodium-ion batteries
Source: Proc Natl Acad Sci U S A. 2024 Jul 25;121(31):e2319193121. doi: 10.1073/pnas.2319193121 (PMC11295058; doi:10.1073/pnas.2319193121)
Supplement: Supplementary file 1 — Appendix 01 (PDF) [file pnas.2319193121.sapp.pdf]

Supporting Information for
Coordination Engineering for Iron-based Hexacyanoferrate as a High-stability Cathode for Sodium−Ion Batteries

Jiang Zhong[a], Lirong Xia[b], Song Chen[a], Zhengwei Zhang[c], Yong Pei[b], Hao Chen[a], Hongtao Sun[d], Jian Zhu[a,e,1], Bingan Lu[a], and Yinghe Zhang[f,1]

[a]State Key Laboratory for Chemo/Biosensing and Chemometrics, College of Chemistry and Chemical Engineering, School of Physics and Electronics, Hunan Key Laboratory of Two-Dimensional Materials, Engineering Research Center of Advanced Catalysis of the Ministry of Education, Hunan University, Changsha 410082, People's Republic of China
[b]Department of Chemistry, Key Laboratory of Environmentally Friendly Chemistry and Applications of Ministry of Education, Xiangtan University, Xiangtan 411105, People's Republic of China
[c]Hunan Key Laboratory of Nanophotonics and Devices, School of Physics and Electronics, Central South University, Changsha 410083, People's Republic of China
[d]The Harold and Inge Marcus Department of Industrial Engineering, The Pennsylvania State University, State College, University Park, PA 16802, USA
[e]Shenzhen Research Institute, Hunan University, Shenzhen 518000, People's Republic of China
[f]School of Civil and Environmental Engineering, Harbin Institute of Technology, Shenzhen Key Laboratory of Advanced Functional Carbon Materials Research and Comprehensive Application, Shenzhen 518055, People's Republic of China
[1] To whom correspondence may be addressed. Email: jzhu@hnu.edu.cn (J. Zhu) and/or zhangyinghe@hit.edu.cn

This PDF file includes:

Figures S1 to S27
Tables S1 to S10
SI References

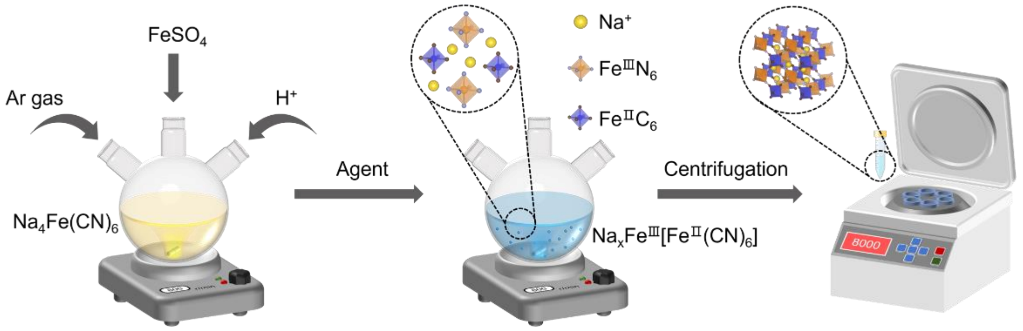

Fig.S1. Schematic illustrations of the synthetic route for all of the NFCN samples.

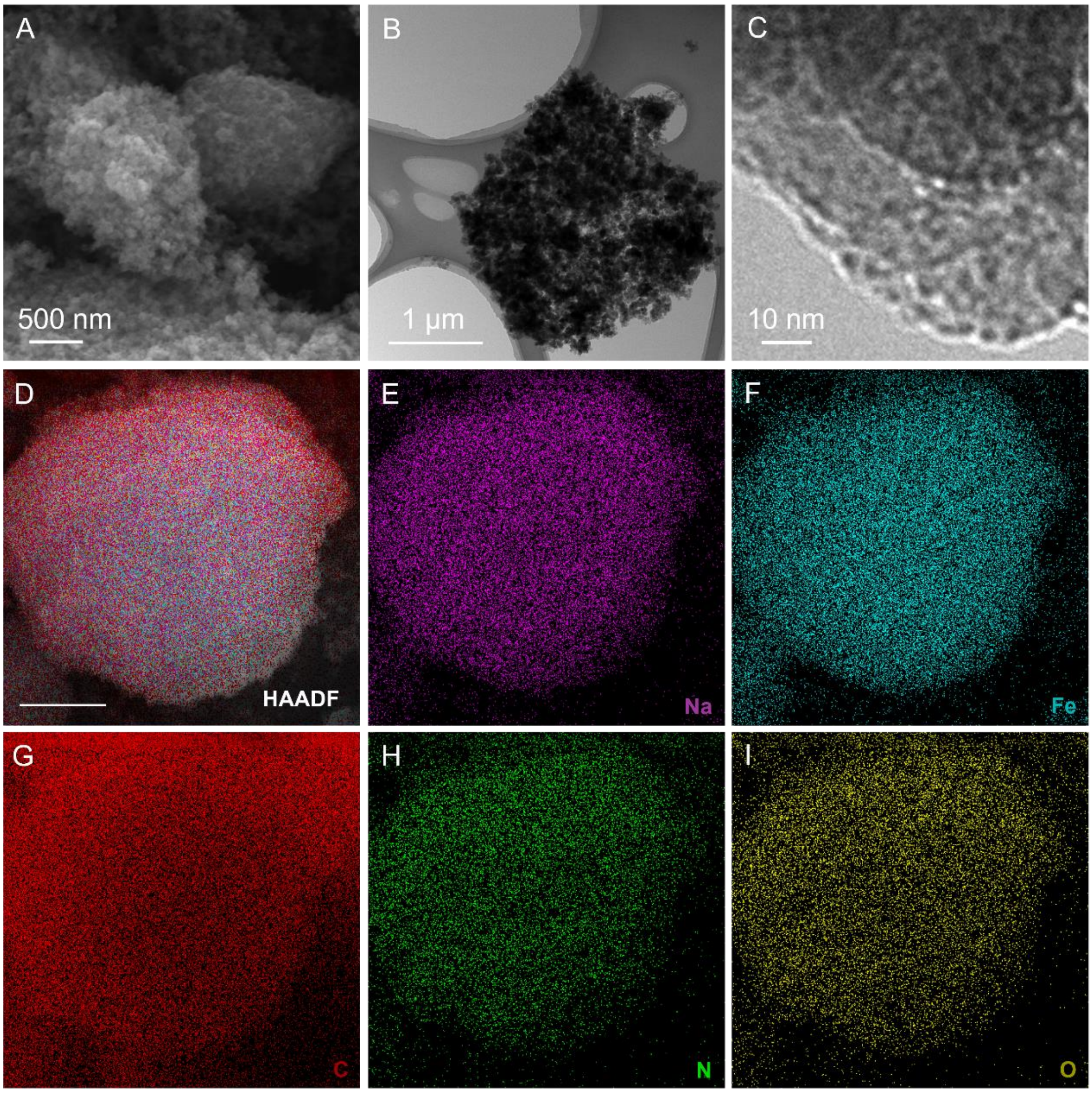

Fig. S2. SEM (A) and TEM (B, C) for NFCN-1 sample. EDS (D to I) mapping of the different elements in the NFCN-1 sample, the scale bar is 2.5 μm.

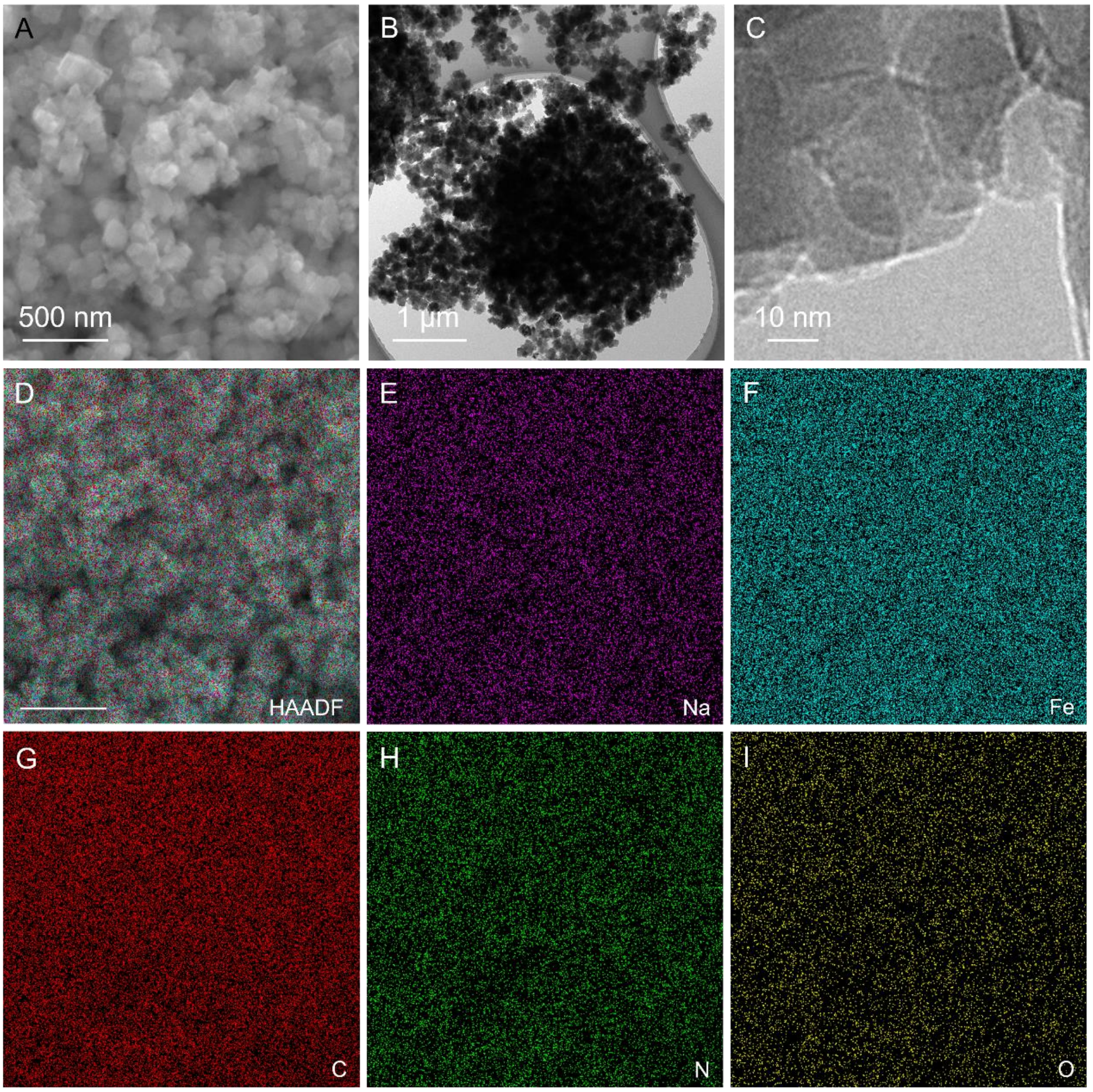

Fig. S3. SEM (A) and TEM (B, C) for NFCN-3 sample. EDS (D to I) mapping of the different elements in the NFCN-3 sample, the scale bar is 1 μm.

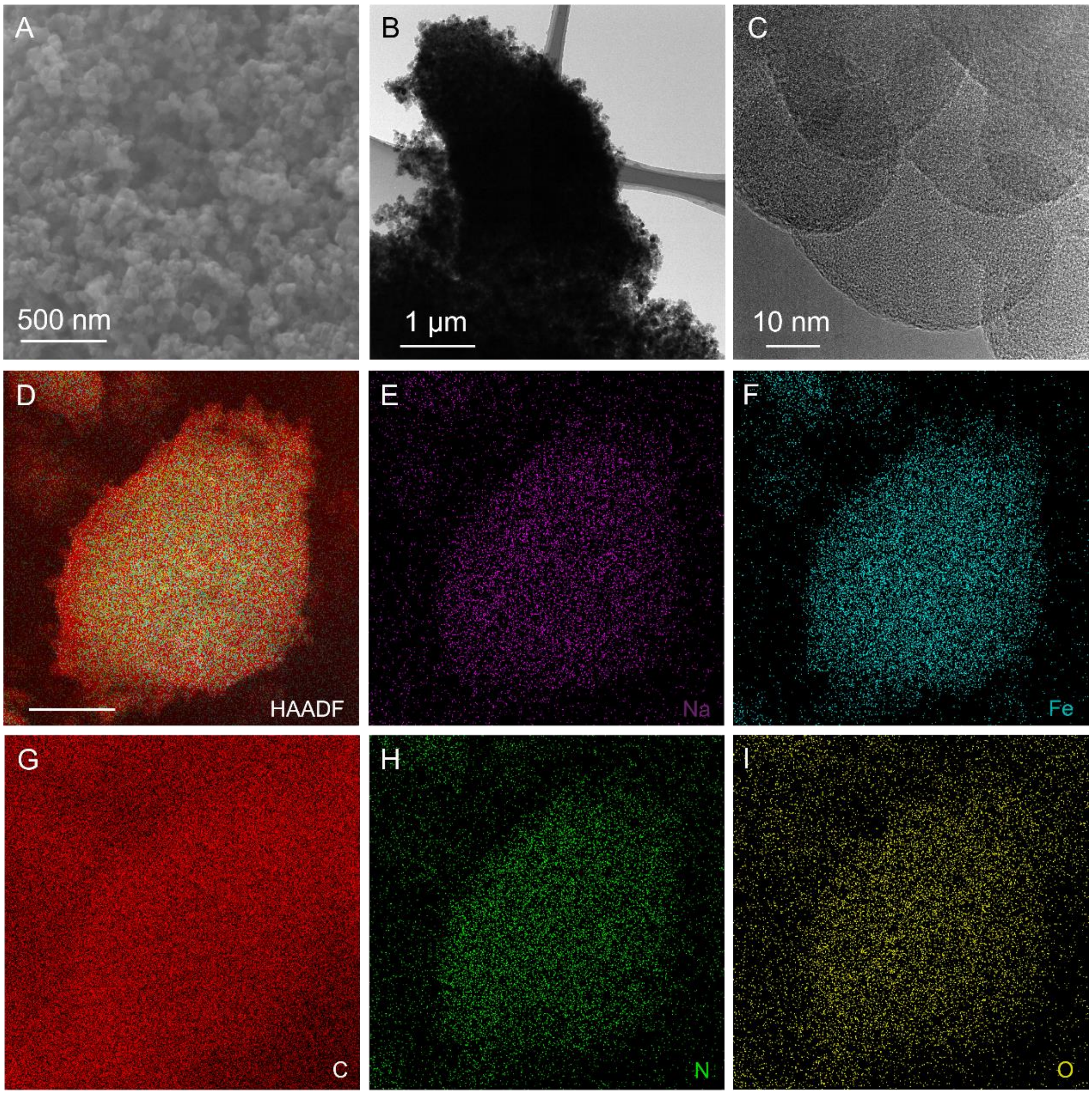

Fig. S4. SEM (A) and TEM (B, C) for NFCN-4 sample. EDS (D to I) mapping of the different elements in the NFCN-4 sample, the scale bar is 1 μm.

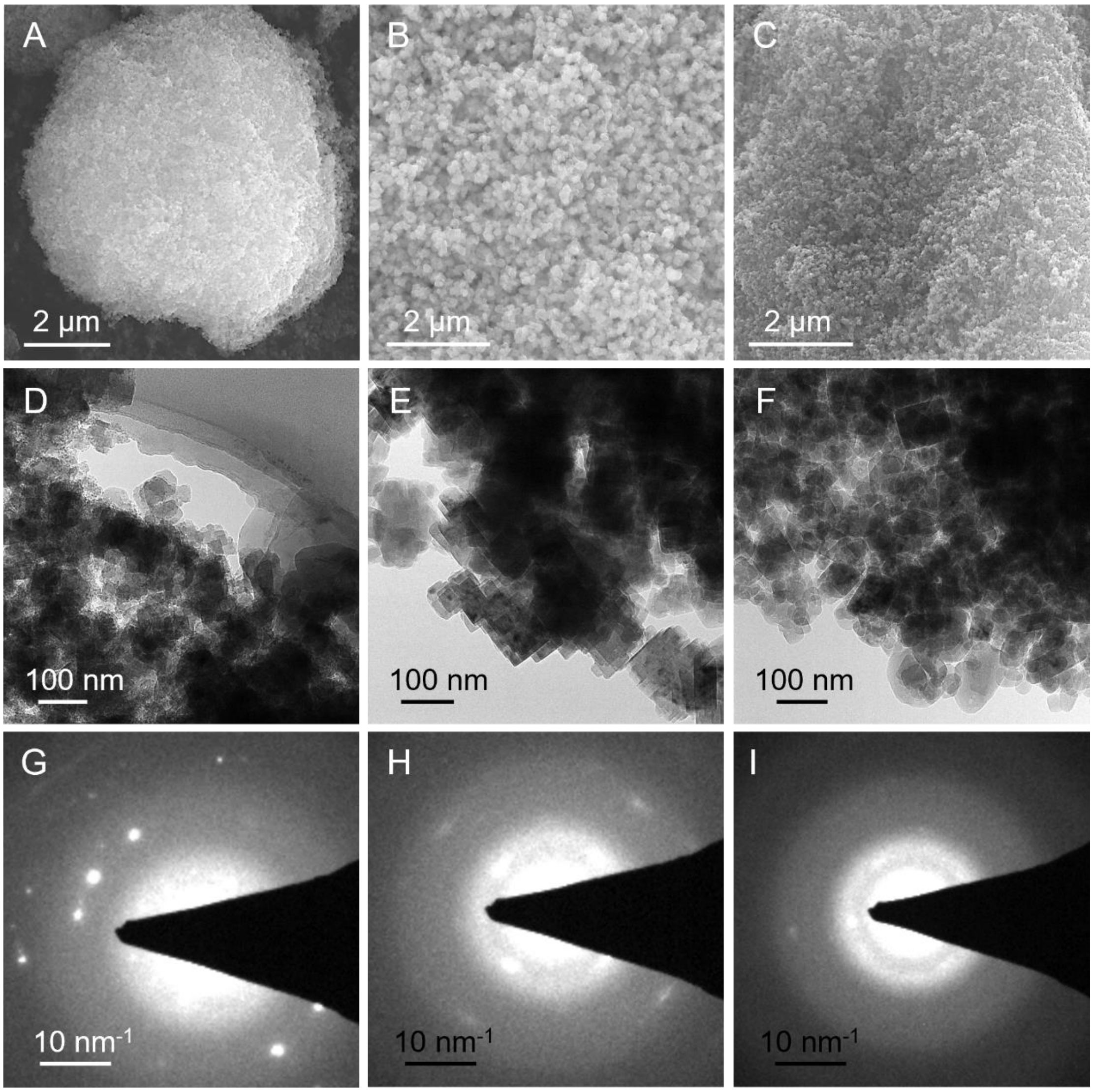

Fig. S5. SEM (A−C), TEM (D−F) and SAED (G−I) for NFCN-1, NFCN-3, and NFCN-4 sample, respectively.

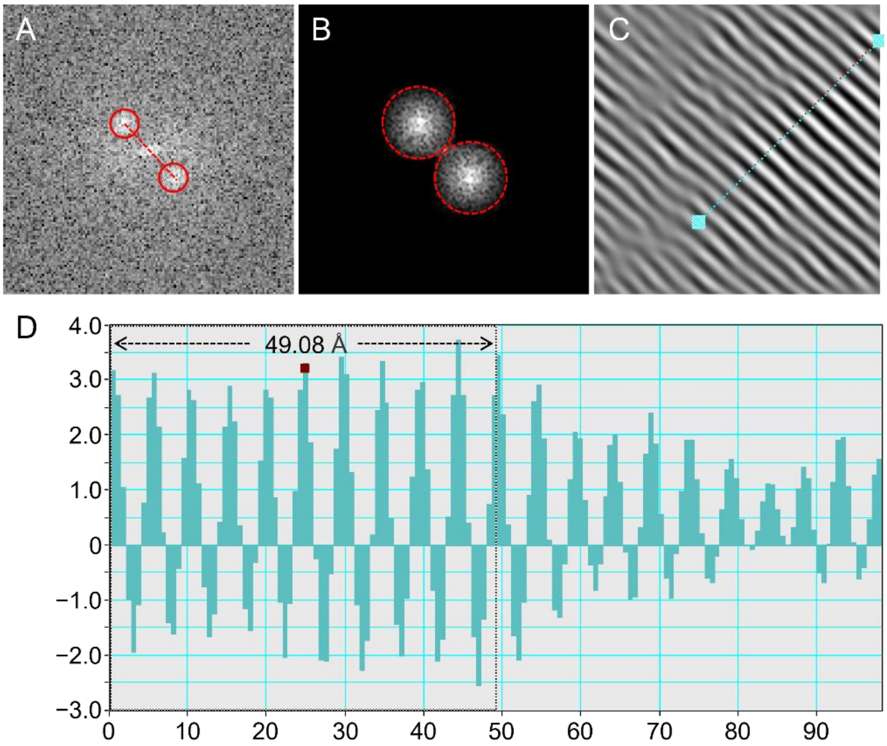

Fig. S6. (A−C) HRTEM with the FFT/ inverse FFT for the NFCN-2 sample. (D) The measurement process of lattice spacing.

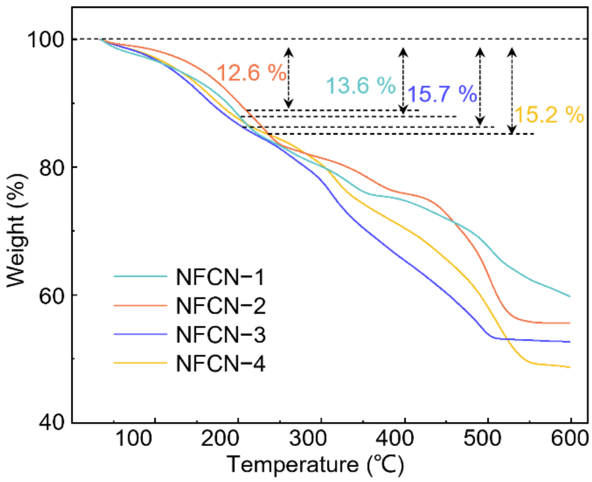

Fig. S7. TGA results for the water content of as-obtained NFCN samples.

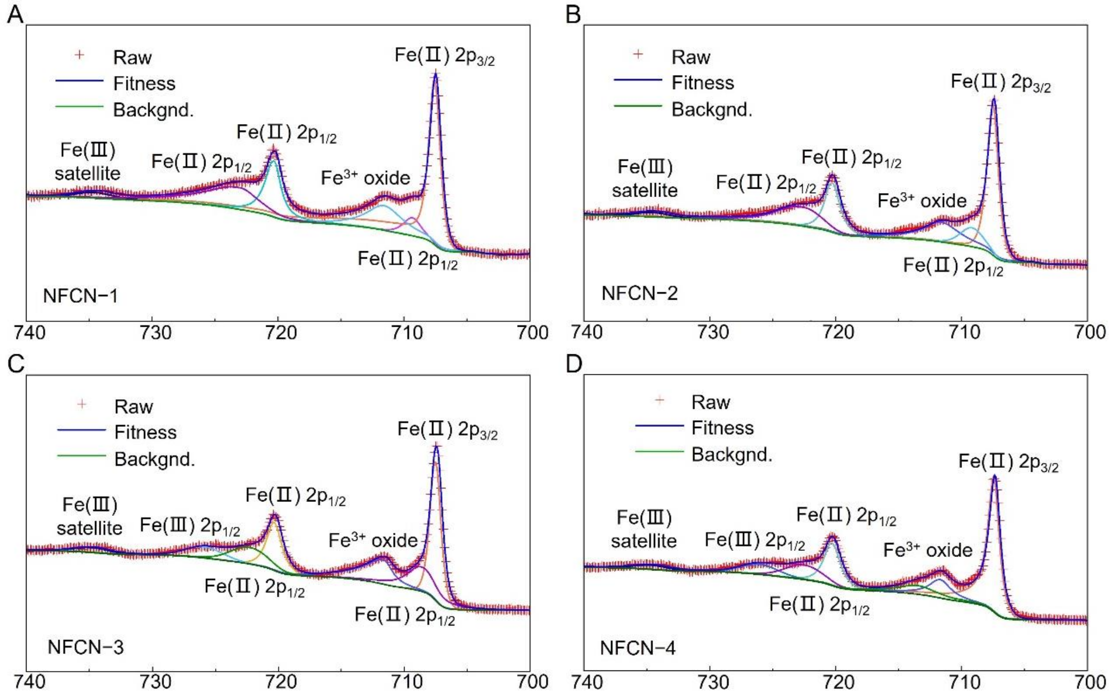

Fig. S8. High-resolution Fe 2p X-ray photoelectron spectroscopy for NFCN-1 (A), NFCN-2 (B), NFCN-3 (C), and NFCN-4 (D) sample.

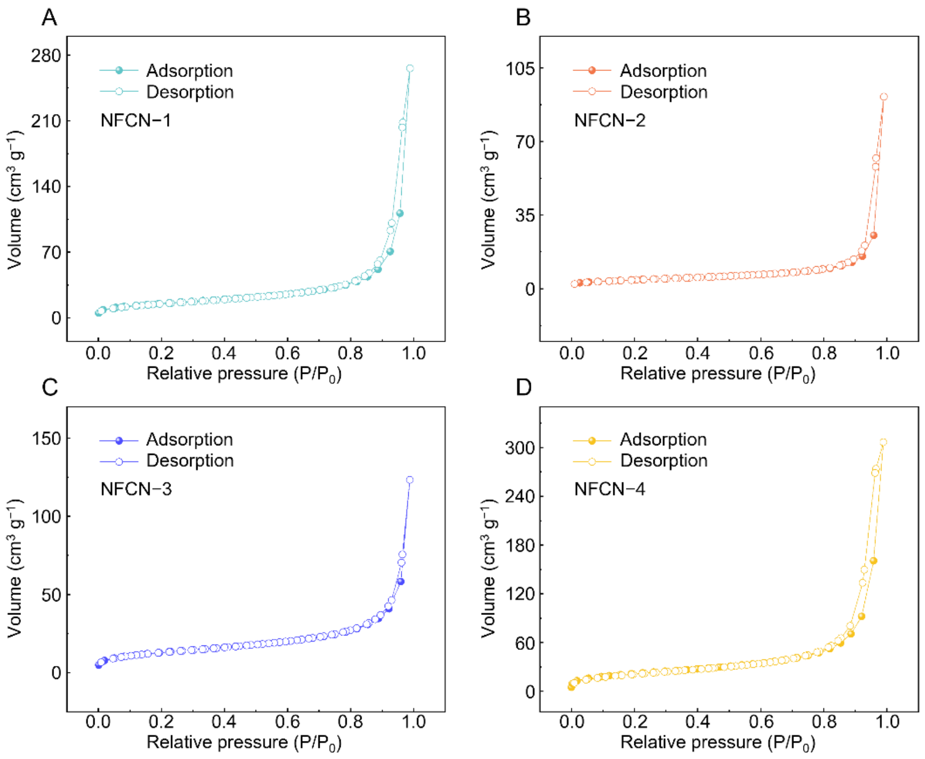

Fig. S9. (A–D) N_2 adsorption–desorption isotherms for all of NFCN samples.

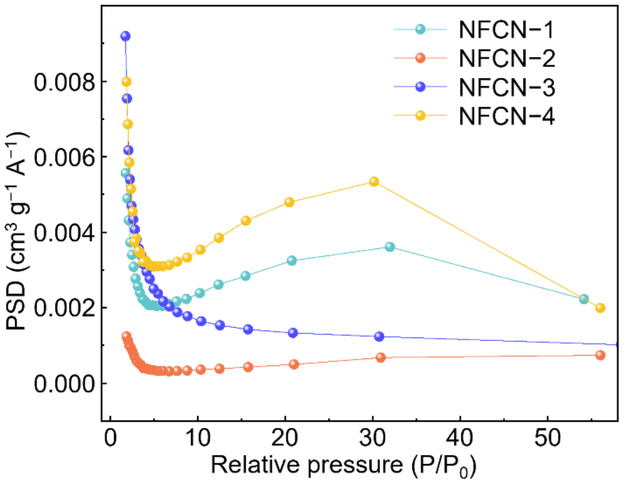

Fig. S10. Pore-size distribution of all NFCN samples.

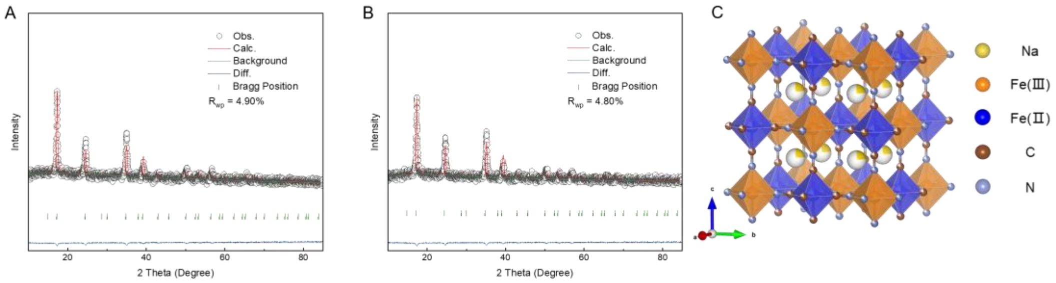

Fig. S11. Rietveld refinement (A for 0 °C and B for 50 °C) and structure (C) results for different synthesis temperature.

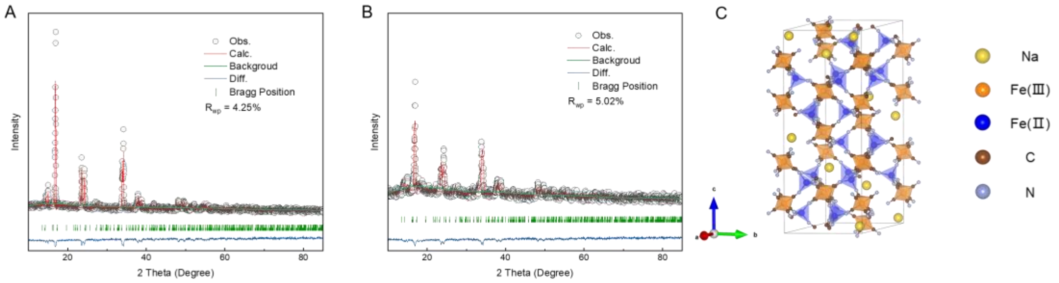

Fig. S12. Rietveld refinement (A for $Na_2C_2O_4$ and B for NaCl) and structure (C) results for different synthesis complexing agents.

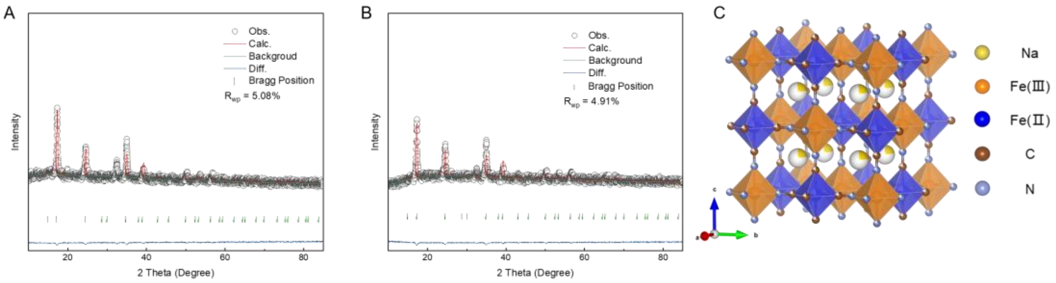

Fig. S13. Rietveld refinement (A for C_2H_5OH and B for $C_2H_4O_2H_2$) and structure (C) results for different solvent.

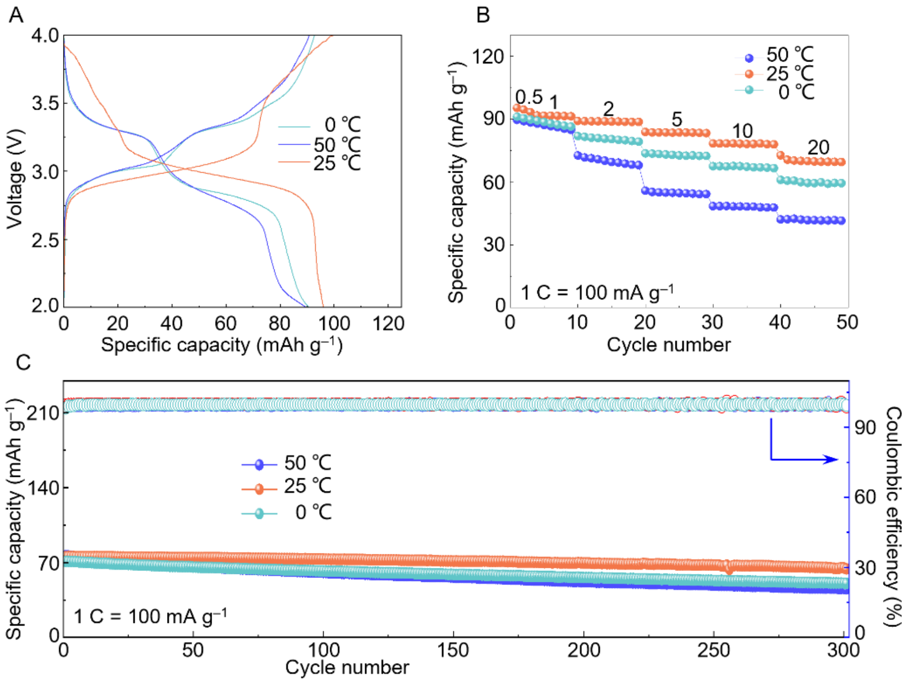

Fig. S14. (A) The initial charge-discharge curves for NFCN-2 material (at the same pH value, chelated agent, and H_2O solvent) at different temperature synthesis condition. (B) Rate performance at different current densities from 50 mA g^{-1} to 2000 mA g^{-1} (0.5−20 C). (C) Long-term cycling at 200 mA g^{-1}.

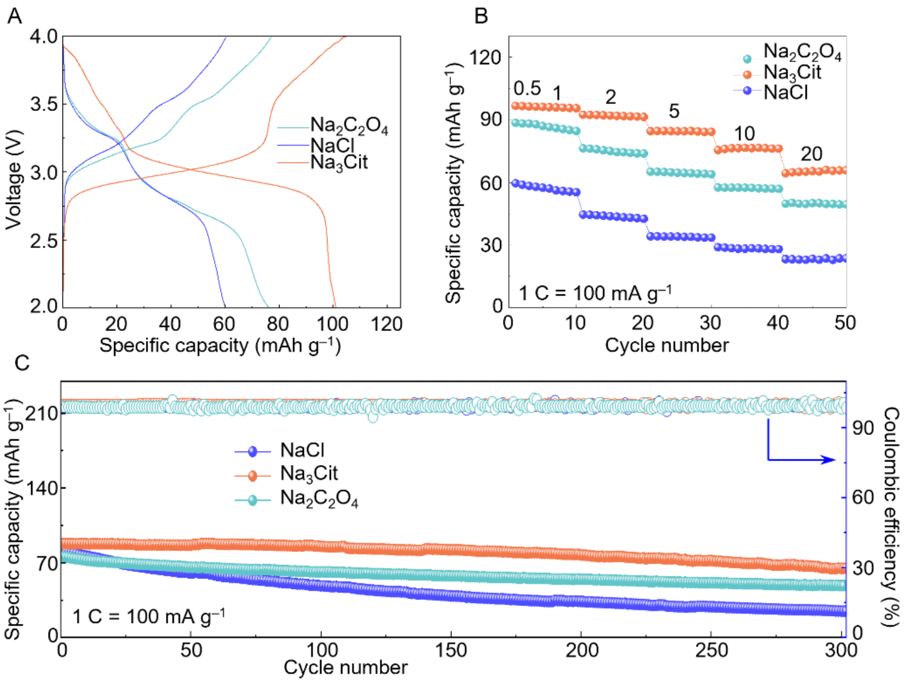

Fig. S15. (A) The initial charge-discharge curves for NFCN-2 material (at the same pH value, temperature, and H_2O solvent) at different complexing agent synthesis condition. (B) Rate performance at different current densities from 50 mA g^{-1} to 2000 mA g^{-1} (0.5−20 C). (C) Long-term cycling at 200 mA g^{-1}.

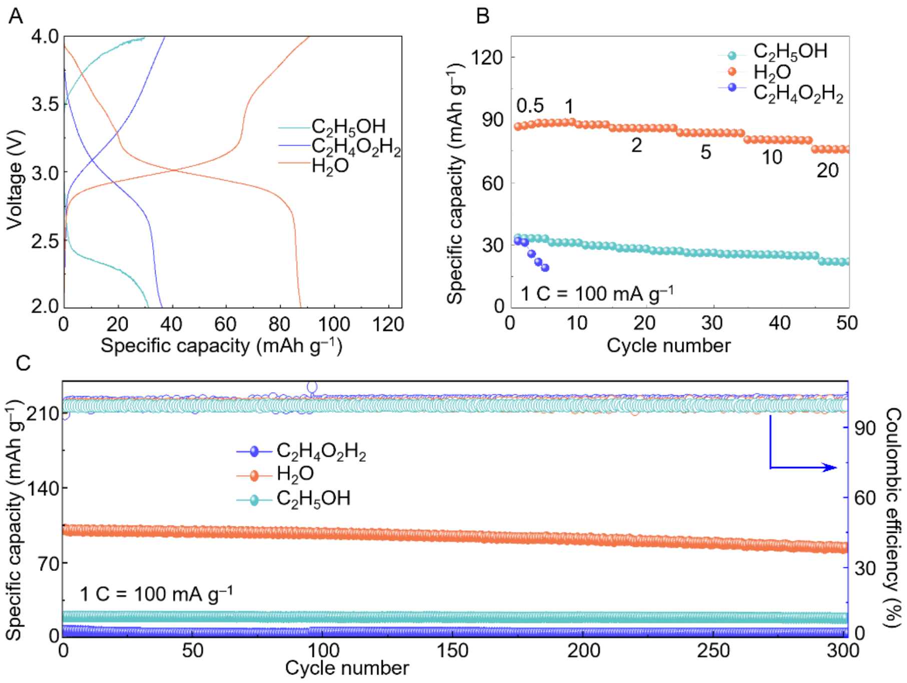

Fig. S16. (A) The initial charge-discharge curves for NFCN-2 material (at the same pH value, chelated agent, and temperature) at different solvent synthesis condition. (B) Rate performance at different current densities from 50 mA g^{-1} to 2000 mA g^{-1} (0.5−20 C). (C) Long-term cycling at 200 mA g^{-1}.

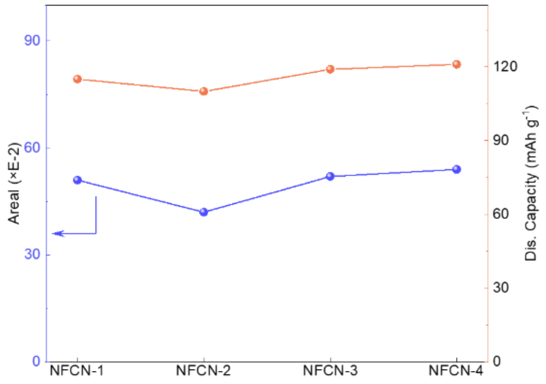

Fig. S17. Integration areal and corresponding discharge capacity for NFCN-1, NFCN-2, NFCN-3, and NFCN-4, respectively.

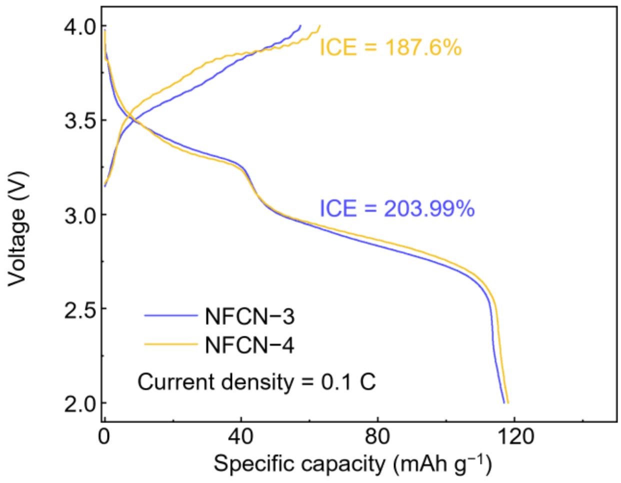

Fig. S18. The initial charge–discharge curves for NFCN-3 and NFCN-4.

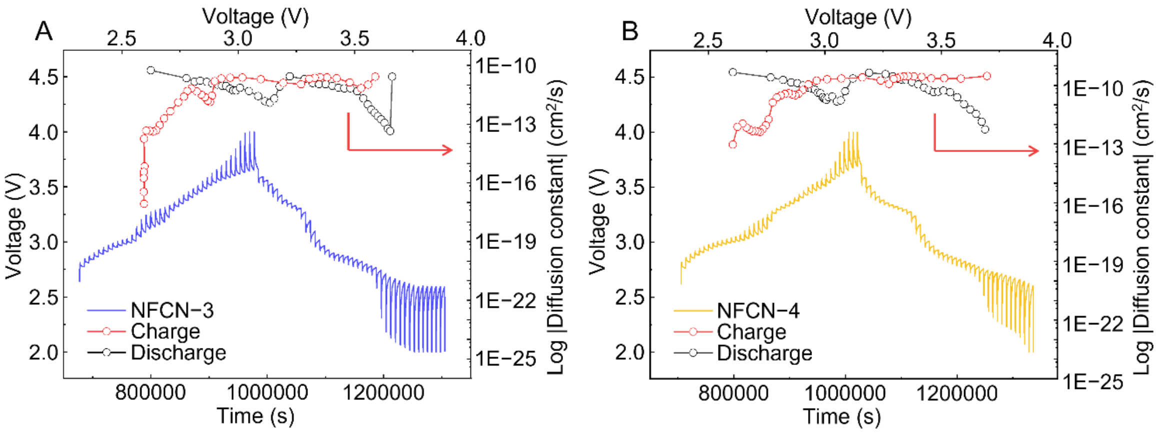

Fig. S19. The GITT curves and calculated diffusion constant of Na^+ during the charging/discharge process of the NFCN-3 (A), and NFCN-4 (B) samples.

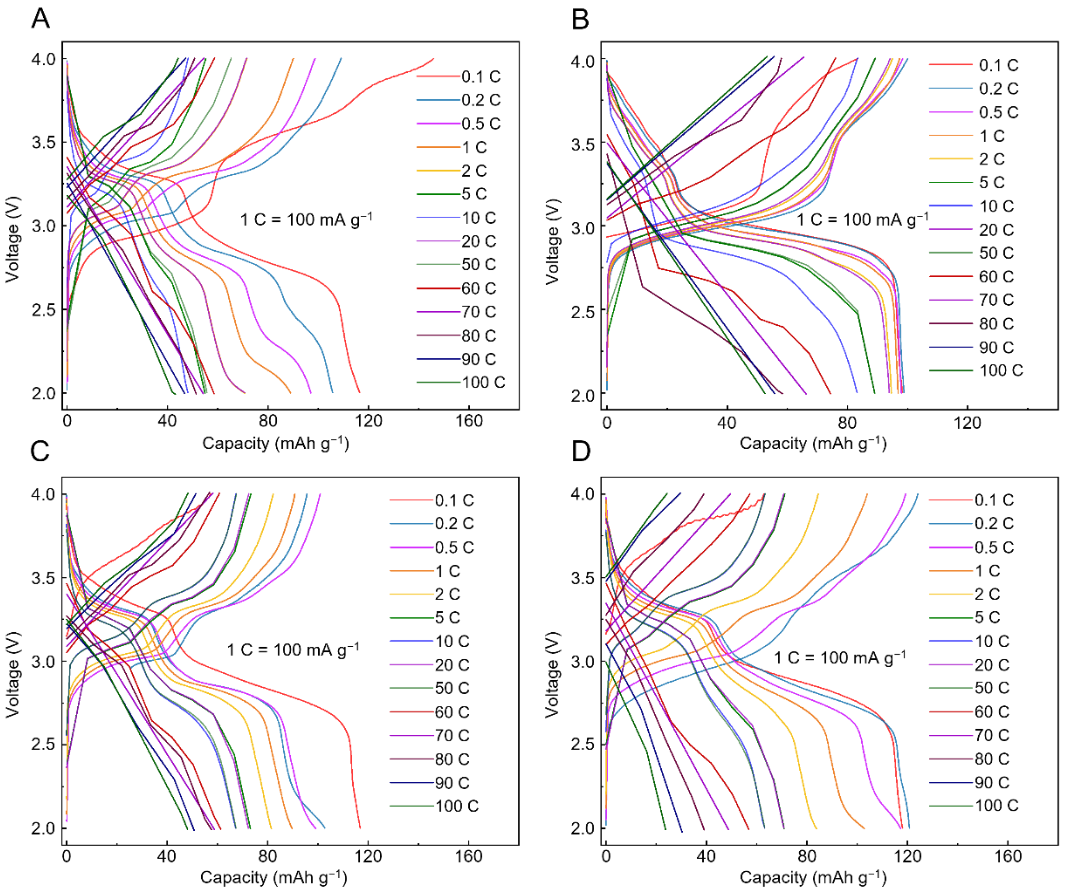

Fig. S20. Electrochemical performances of NFCN-1 (A), NFCN-2 (B), NFCN-3 (C), and NFCN-4 (D) sample.

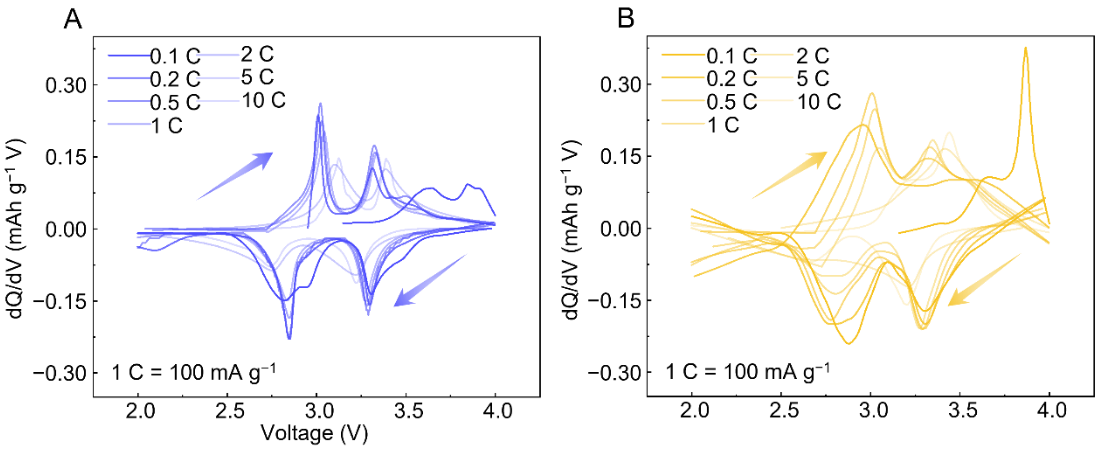

Fig. S21. The dQ/dV curves of NFCN-3 (A) and NFCN-4 (B) at different rates in 2.0–4.0 V.

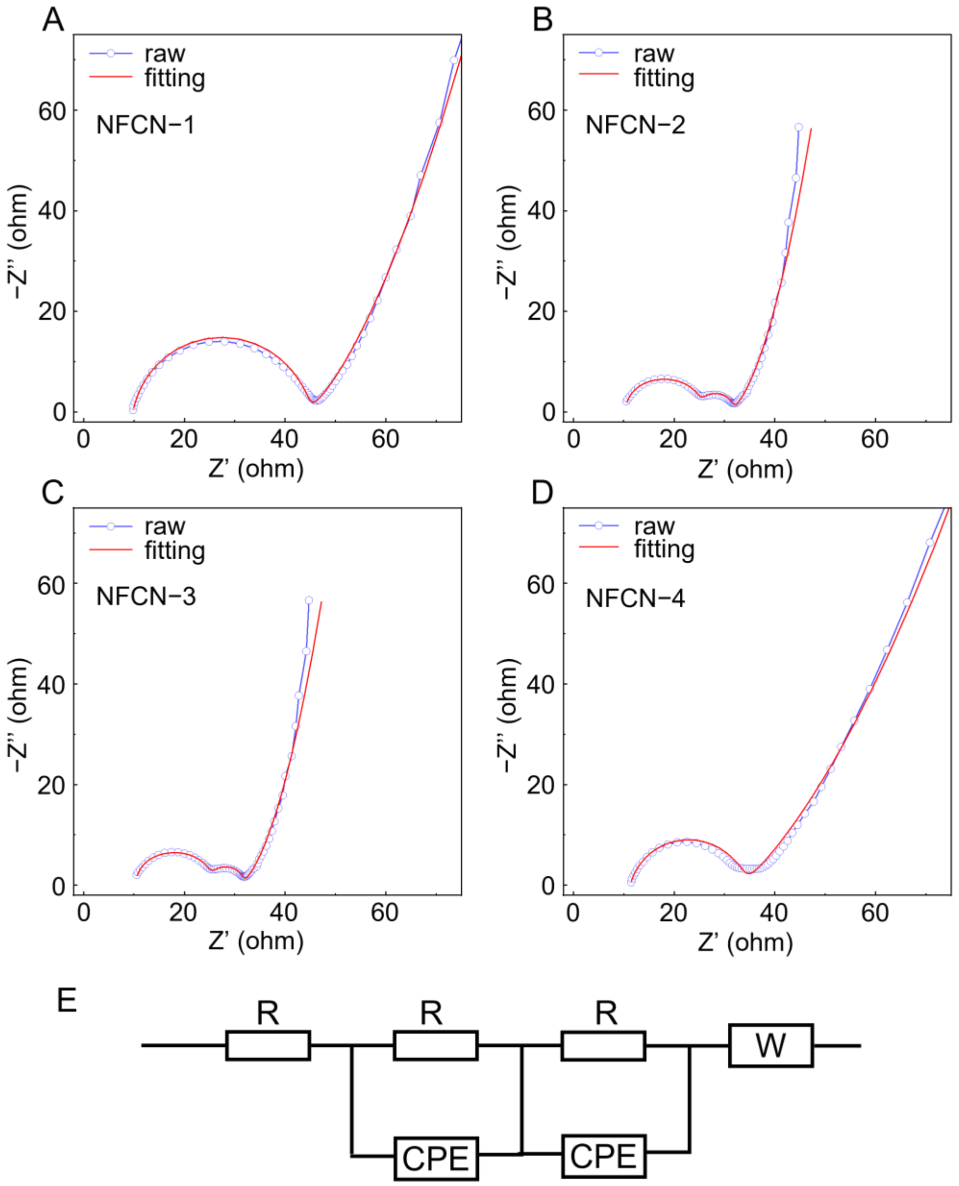

Fig. S22. Nyquist plots of the NFCN-1 (A), NFCN-2 (B), NFCN-3 (C), and NFCN-4 (D) sample. (E) the equivalent circuit modeling the EIS spectra.

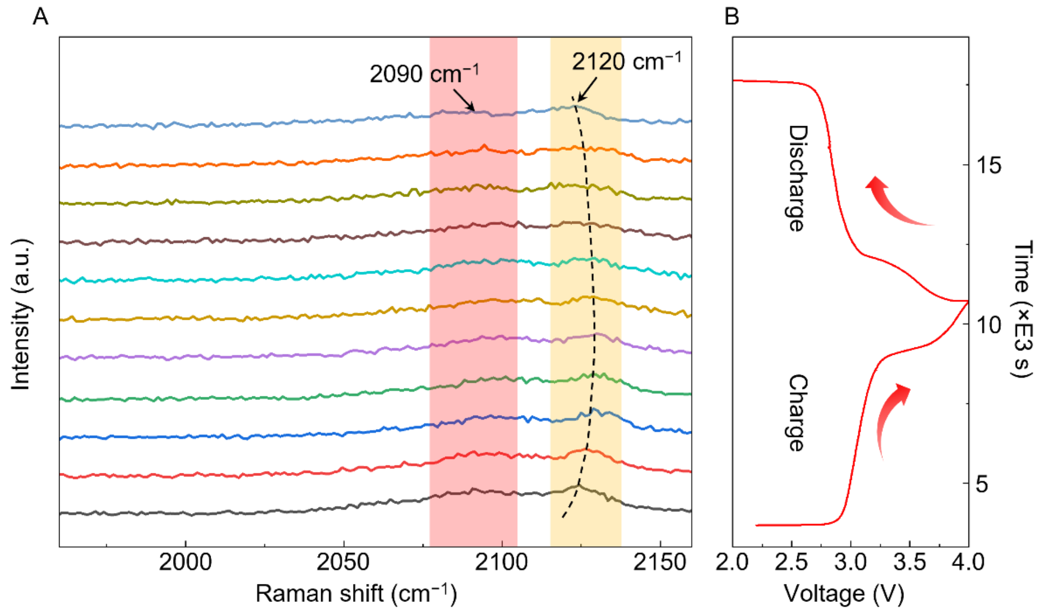

Fig. S23. *In-situ* Raman line spectra (A) and corresponding time/charge voltage profile at a current density of 50 mA g^{-1} (B).

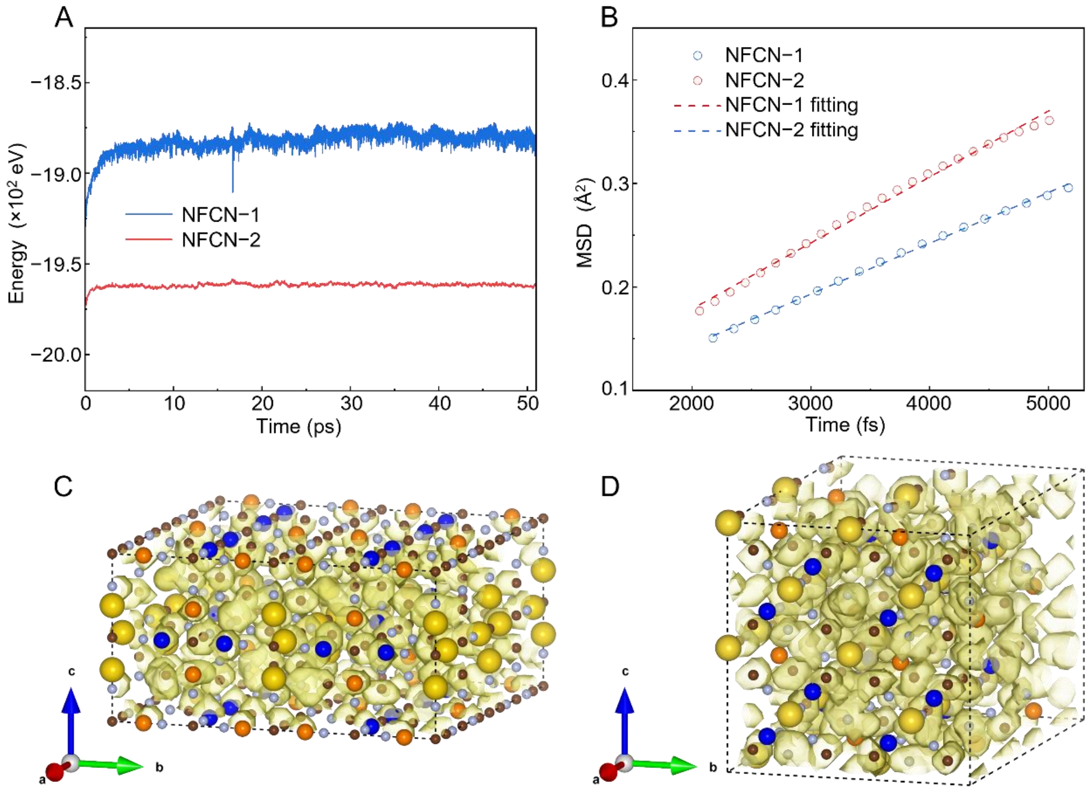

Fig. S24. Theoretical study of the stability and the diffusion pathways of Na^+ for NFCN-1 and NFCN-2 from AIMD simulation at 900K. (**A**) The energy distribution for NFCN-1 and NFCN-2 systems. (**B**) The MSD of Na^+ as a function of time for NFCN-1 and NFCN-2 systems. The Na^+ probability density distribution of the NFCN-1 (**C**) and NFCN-2 (**D**) framework (the electron density scale is 0.0372 a.u.).

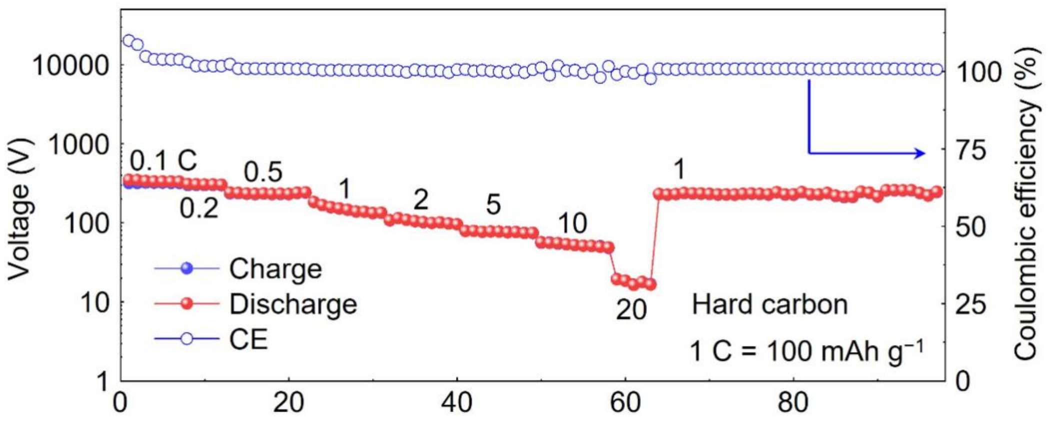

Fig. S25. The rate and cycling capability of the commercial hard carbon electrode.

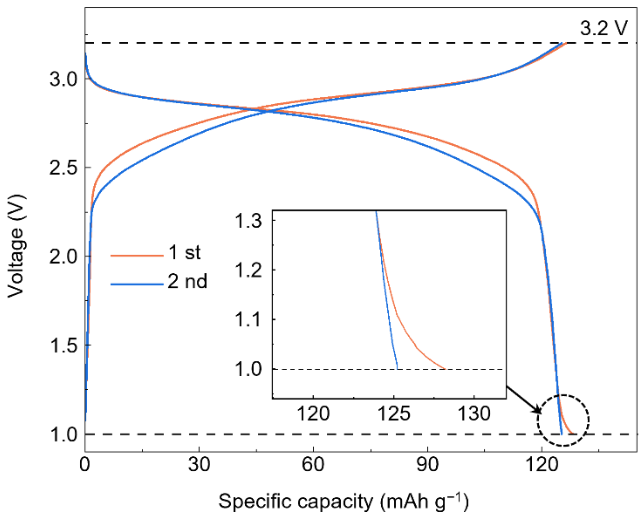

Fig. S26. Charge/discharge curves during 1st and 2nd cycle at 0.1 C of the full cell.

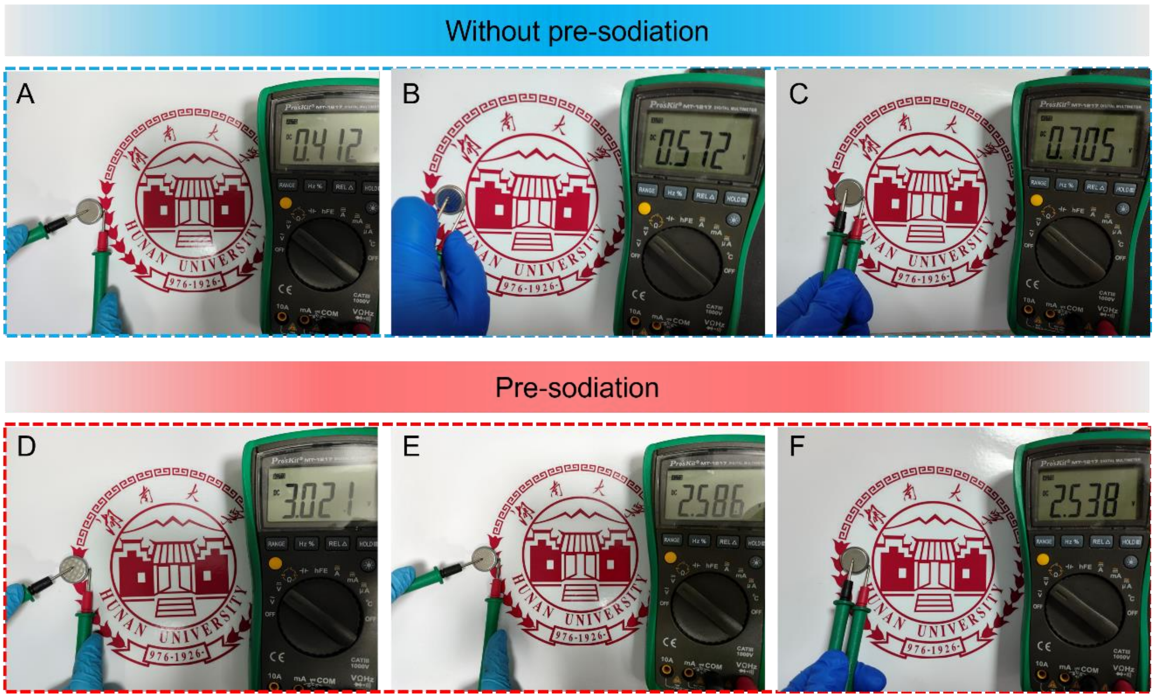

Fig. S27. The value of the open circuit potential for NFCN-2|HC full-cell. (A to C) without pre-sodiation. (D to F) with pre-sodiation.

Table S1. The formulae of PB-1, PB-2, PB-3, PB-4, and PB-5 and pH values of different mixed solvents for the preparation of the corresponding sample.

Sample	pH	Formula
NFCN-1	4.53	$Na_{0.97}Fe[Fe(CN)_6]\cdot 1.36H_2O$
NFCN-2	3.75	$Na_{1.27}Fe[Fe(CN)_6]\cdot 1.26H_2O$
NFCN-3	2.98	$Na_{1.15}Fe[Fe(CN)_6]\cdot 1.57H_2O$
NFCN-4	1.03	$Na_{1.21}Fe[Fe(CN)_6]\cdot 1.52H_2O$

Table S2. Detailed lattice paraments for all the NFCN samples after Rietveld refinement.

Sample	Space group	a/Å	b/Å	c/Å	alpha	beta	gamma
NFCN-1	$Fm\overline{3}m$	10.28760	10.28760	10.28760	90.0000	90.0000	90.0000
NFCN-2	*P21/n*	10.36830	7.35250	7.30180	90.0000	91.5810	90.0000
NFCN-3	$Fm\overline{3}m$	10.22540	10.22540	10.22540	90.0000	90.0000	90.0000
NFCN-4	$Fm\overline{3}m$	10.22080	10.22080	10.22080	90.0000	90.0000	90.0000

Table S3. Detailed structure information on the NFCN-1 samples after Rietveld refinement.

Atoms	x	y	z	Occupation
Fe	0.5	1	0.5	1
Fe	0.5	0.5	0.5	1
Na	0.75	0.75	0.75	1
O	0.5	1	0.849	0.219
N	0.5	0.802	0.5	1
C	0.5	0.727	0.5	1

Table S4. Detailed structure information on the NFCN-2 samples after Rietveld refinement.

Atoms	x	y	z	Occupation
Fe	0.5	0.5	0.5	1
Fe	0.5	0	1	1
N	0.501	0.302	0.711	1
N	0.313	0.53	0.512	1
N	0.522	0.271	0.243	1
C	0.494	0.208	0.843	1
C	0.198	0.492	0.511	1
C	0.522	0.08	0.351	1
Na	0.27	0.478	0.003	0.793
O	0.256	0.312	0.336	0.829

Table S5. ICP-OES results on Fe and Na for NFCN-1, NFCN-2, NFCN-3, and NFCN-4 samples.

Sample	Na (ppm)	Fe (ppm)
NFCN-1	96717	329810
NFCN-2	126573	347323
NFCN-3	115038	159324
NFCN-4	120678	172261

Table S6. Material parameters of various NFCN electrodes.

Sample	BET surface ($m^2\ g^{-1}$)	Volume ($cm^3\ g^{-1}$)
NFCN-1	55.8346	0.4092
NFCN-2	15.1236	0.1408
NFCN-3	45.9758	0.1885
NFCN-4	78.3562	0.4704

Table S7. Detailed lattice paraments for all the NFCN samples after Rietveld refinement.

T	Space group	a/Å	b/Å	c/Å	alpha	beta	gamma
0 °C	$Fm\bar{3}m$	10.26727	10.26727	10.26727	90.0000	90.0000	90.0000
25 °C	*P21/n*	10.36830	7.35250	7.30180	90.0000	91.5810	90.0000
50 °C	$Fm\bar{3}m$	10.23416	10.23416	10.23416	90.0000	90.0000	90.0000

Table S8. Detailed lattice paraments for all the NFCN samples after Rietveld refinement.

Chelate	Space group	a/Å	b/Å	c/Å	alpha	beta	gamma
$Na_2C_2O_4$	$R\bar{3}c$	12.46900	12.46900	32.92000	90.0000	90.0000	120.0000
NaCl	$R\bar{3}c$	12.45470	12.46110	32.87400	90.0000	90.0000	120.0000
Na_3Cit	*P21/n*	10.36830	7.35250	7.30180	90.0000	91.5810	90.0000

Table S9. Detailed lattice paraments for all the NFCN samples after Rietveld refinement.

Solvent	Space group	a/Å	b/Å	c/Å	alpha	beta	gamma
C_2H_5OH	$Fm\bar{3}m$	10.26727	10.26727	10.26727	90.0000	90.0000	90.0000
H_2O	*P21/n*	10.36830	7.35250	7.30180	90.0000	91.5810	90.0000
$C_2H_4O_2H_2$	$Fm\bar{3}m$	10.26741	10.26741	10.26741	90.0000	90.0000	90.0000

Table S10. Summary of the published full cell for SIBs based on the PBAs cathodes.

**Cathode**	**Anode**	**Voltage range (V)**	**Current density (mA g^{-1})**	**Capacity decay rate per cycle**	**Cycles**
**NFCN-2 (this work)**	**hard carbon**	**1.0–3.2**	**500**	**0.0128%**	**over 1000**
$Na_{0.22}Ni[Fe(CN)_6]$[1]	hard carbon	1.8–3.7	85	0.100%	250
$Na_{0.647}Fe[Fe(CN)_6]$[2]	hard carbon	2.0–4.0	100	0.170%	25
$Na_2FeCuFe(CN)_6$[3]	$NaTi_2(PO_4)_3$	0.5–2.5	50	0.037%	400
$Na_{2-x}FeFe(CN)_6$[4]	hard carbon	1.0–3.2	170	0.078%	1000
$Na_{1.92}Fe_2(CN)_6$[5]	hard carbon	1.0–3.5	10	0.560%	50
$Na_{1.68}N_iCo[Fe(CN)_6]$[6]	$NaTi_2(PO_4)_3$	0.5–2.2	150	0.200%	300
$Na_xFeFe(CN)_6$[7]	FeO_x	0.5–3.0	250	0.030%	400
$Na_2MnFe(CN)_6$[8]	hard carbon	1.5–3.8	100	0.298%	30

Note:
[1] bold indicates the excellent full-cell performance (better than that of other electrodes);
[2] the capacity is based on the mass loading for the cathode.

SI References

1. Y. Xu, *et al.*, Crystallization-induced ultrafast Na-ion diffusion in nickel hexacyanoferrate for high-performance sodium-ion batteries, *Nano Energy* 67, 104250 (2020).
2. Y. Jiang, *et al.*, Prussian blue@C composite as an ultrahigh-rate and long-life sodium-ion battery cathode, *Adv. Funct. Mater.* 26, 5315-5321 (2016).
3. Z. Wang, *et al.*, Ion-exchange synthesis of high-energy-density Prussian blue analogues for sodium ion battery cathodes with fast kinetics and long durability, *J. Power Sources* 436, 226868 (2019).
4. W. Wang, *et al.*, Reversible structural evolution of sodium-rich rhombohedral Prussian blue for sodium-ion batteries, *Nat. Commun.* 11, 1-9 (2020).
5. L. Wang, *et al.*, Rhombohedral Prussian white as cathode for rechargeable sodium-ion batteries, *J. Am. Chem. Soc.* 137, 2548-2554 (2015).
6. J. Peng, et al., A dual-insertion type sodium-ion full cell based on high-quality ternary-metal Prussian blue analogs, *Adv. Energy. Mater.* 8, 1702856 (2018).
7. H. Ye, *et al.*, Iron-based sodium-ion full batteries, *J. Mater. Chem. A* 4, 1754-1761 (2016).
8. J. Song, *et al.*, Removal of interstitial H_2O in hexacyanometallates for a superior cathode of a sodium-ion battery, *J. Am. Chem. Soc.* 137, 2658-2664 (2015).
